# Supplementary material for: The relationship between early life urbanicity and depression in late adulthood: evidence from the Survey of Health, Ageing and Retirement in Europe
Source: BMJ Open. 2019 Sep 5;9(9):e028090. doi: 10.1136/bmjopen-2018-028090 (PMC6731892; doi:10.1136/bmjopen-2018-028090)
Supplement: Supplementary data [file bmjopen-2018-028090supp001.pdf]

TABLE A1
Item missingness: summary (all individuals in both waves, n=25,341)

Variable	Missing count	Present count
EURO-D	487	24,854
Childhood urban residence	1,715	23,626
Age	716	24,625
Childhood maths performance	859	24,482
Childhood language performance	927	24,414
Childhood self-reported health	189	25,152
Parent heavy drinker	103	25,238
Deprivation score	1,172	24,169
Biological Father Present	506	24,835
Biological Mother Present	506	24,835
Stepfather Present	506	24,835
Stepmother Present	506	24,835
Equivalised HH income	444	24,897
Current urban residence	8,882	16,459

TABLE A2

Principal component analysis for childhood SES

	Component			
	1st	2nd	3rd	4th
Number of Rooms	0.4073	0.8637	-0.2968	-0.0111
Number of Books (grouping)	0.5651	-0.1752	0.2367	0.7707
Occupation of Main Breadwinner (ranked)	-0.4758	0.4695	0.7042	0.2394
Number of Facilities	0.537	-0.0546	0.6	-0.5905

TABLE A3

Urbanicity of childhood residence and of current residence (all values percentages)

		Current residence					
		1	2	3	4	5	Total
Childhood residence	1	37.65	21.28	15.13	11.31	14.63	100
	2	17.19	39.64	14.03	14.84	14.30	100
	3	14.39	16.20	38.87	16.81	13.73	100
	4	11.14	12.62	20.40	38.10	17.75	100
	5	9.31	9.05	15.80	21.98	43.85	100
	Total	15.23	15.04	19.74	21.89	28.11	100

Key

1: A big city

2: The suburbs or outskirts of a big city

3: A large town

4: A small town

5: A rural area or village

TABLE A4

Association Between Stage of Childhood Urbanicity and Late-Adulthood Depression Outcomes

	OLS			Logit, average marginal effects		
	(1)	(2)	(3)	(4)	(5)	(6)
Age	0-5	6-10	11-15	0-5	6-10	11-15
Childhood living environment (omitted: rural area or village)						
Big city	0.0913**	0.104**	0.124***	0.0193**	0.0215**	0.0234***
	(0.0437)	(0.0440)	(0.0439)	(0.00889)	(0.00897)	(0.00893)
Suburbs or outskirts of a big city	0.173***	0.159***	0.194***	0.0234**	0.0209*	0.0152
	(0.0577)	(0.0562)	(0.0554)	(0.0117)	(0.0114)	(0.0114)
Large town	0.0706	0.0781*	0.0977**	0.00527	0.00831	0.00729
	(0.0439)	(0.0437)	(0.0434)	(0.00915)	(0.00912)	(0.00905)
Small town	-0.0119	0.0165	0.0187	-0.00171	0.00242	-0.00300
	(0.0410)	(0.0410)	(0.0409)	(0.00874)	(0.00874)	(0.00875)
	-	-	-	-	-	
Male	0.845***	0.845***	0.845***	0.121***	0.121***	-0.121***
	(0.0287)	(0.0287)	(0.0287)	(0.00583)	(0.00583)	(0.00583)
Observations	20,400	20,400	20,400	20,400	20,400	20,400

*Notes: Constant not reported. */**/*** indicate statistical significance at the 10%/5%/1% level, standard errors between brackets.*

All models contain controls for age-period-cohort, country fixed effects and additional childhood controls & contemporaneous income.
