# Supplementary material for: The relationship between early life urbanicity and depression in late adulthood: evidence from the Survey of Health, Ageing and Retirement in Europe
Source: BMJ Open. 2019 Sep 5;9(9):e028090. doi: 10.1136/bmjopen-2018-028090 (PMC6731892; doi:10.1136/bmjopen-2018-028090)
Supplement: Author's manuscript [file bmjopen-2018-028090.draft_revisions.pdf]

BMJ Open is committed to open peer review. As part of this commitment we make the peer review history of every article we publish publicly available.

When an article is published we post the peer reviewers' comments and the authors' responses online. We also post the versions of the paper that were used during peer review. These are the versions that the peer review comments apply to.

The versions of the paper that follow are the versions that were submitted during the peer review process. They are not the versions of record or the final published versions. They should not be cited or distributed as the published version of this manuscript.

BMJ Open is an open access journal and the full, final, typeset and author-corrected version of record of the manuscript is available on our site with no access controls, subscription charges or pay-per-view fees (http://bmjopen.bmj.com).

If you have any questions on BMJ Open's open peer review process please email info.bmjopen@bmj.com

BMJ Open

The Relationship Between Early Life Urbanicity and Depression in Late-Adulthood: Evidence from the Survey of Health, Ageing and Retirement in Europe

Journal:	*BMJ Open*
Manuscript ID	bmjopen-2018-028090
Article Type:	Research
Date Submitted by the Author:	21-Nov-2018
Complete List of Authors:	Howdon, Daniel; University of Leeds Leeds Institute of Health Sciences, Academic Unit of Health Economics<br>Mierau, Jochen; Rijksuniversiteit Groningen Faculteit Economie en Bedrijfskunde, Economics, Econometrics and Finance<br>Liew, Samuel; Copenhagen Business School
Keywords:	MENTAL HEALTH, HEALTH ECONOMICS, PUBLIC HEALTH

SCHOLARONE™
Manuscripts

The Relationship Between Early Life Urbanicity and Depression in Late-Adulthood: Evidence from the Survey of Health, Ageing and Retirement in Europe

Daniel Howdon *, [1, 2] Jochen O. Mierau [2, 3, 4] Samuel K. M. Liew

* Corresponding author. Academic Unit of Health Economics, Leeds Institute of Health Sciences, Level 10, Worsley Building, Clarendon Way, Leeds, LS2 9NL, United Kingdom. d.howdon@leeds.ac.uk

1: Academic Unit of Health Economics, Leeds Institute of Health Sciences, University of Leeds, UK

2: Department of Economics, Econometrics and Finance, Faculty of Economics and Business, University of Groningen, Groningen, Netherlands

3: NETSPAR, Tilburg, Netherlands

4: Aletta Jacobs School of Public Health, University of Groningen, Groningen, Netherlands.

Word count: 3509

For peer review only

Authorship statement:

SL carried out initial empirical estimation and wrote the first draft as a postgraduate thesis under the supervision of DH and JM. DH carried out re-estimation and estimation of additional model specifications, and substantially redrafted the paper. JM and SL made further redrafting contributions to the paper. All authors approved the final draft.

Data sharing:

Data is available by application at http://shareproject.org.

Acknowledgements:

This paper uses data from SHARE Waves 2 and 3 (SHARELIFE) (DOIs: 10.6103/SHARE.w2.610, 10.6103/SHARE.w3.610), see Börsch-Supan et al. (2013) for methodological details. The SHARE data collection has been primarily funded by the European Commission through FP5 (QLK6-CT-2001-00360), FP6 (SHARE-I3: RII-CT-2006-062193, COMPARE: CIT5-CT-2005-028857, SHARELIFE: CIT4-CT-2006-028812) and FP7 (SHARE-PREP: N°211909, SHARE-LEAP: N°227822, SHARE M4: N°261982). Additional funding from the German Ministry of Education and Research, the Max Planck Society for the Advancement of Science, the U.S. National Institute on Aging (U01_AG09740-13S2, P01_AG005842, P01_AG08291, P30_AG12815, R21_AG025169, Y1-AG-4553-01, IAG_BSR06-11, OGHA_04-064, HHSN271201300071C) and from various national funding sources is gratefully acknowledged (see www.share-project.org).

We gratefully acknowledge the insightful comments of two anonymous referees.

Ethics:

An ethics statement related to SHARE/SHARELIFE, the only data on which we rely for this paper, is available at http://www.share-project.org/fileadmin/pdf_documentation/SHARE_ethics_approvals.pdf.

Competing interests:

None to declare

Funding: This project was supported by the Investeringsagenda of the University of Groningen.

Patient and Public Involvement: no patient involvement.

For peer review only - http://bmjopen.bmj.com/site/about/guidelines.xhtml

Abstract

Objectives: We aim to study the association of childhood urbanicity with depressive symptoms in late adulthood.

Design, setting & participants: We use data drawn from 20,400 respondents from the Survey on Health, Ageing and Retirement in Europe, a representative sample of the 50+ population in 13 European countries.

Outcomes & analysis: Childhood urbanicity is determined using self-reports of the respondents' circumstances at age 10, and late-adulthood depression using the EURO-D scale. We condition on circumstances early in life as well as later in life, most importantly late-adulthood urbanicity. We estimate the associations using linear regression models and limited dependent variable models.

Results: A pooled regression of both men and women suggests childhood urbanicity is associated non-monotonically with depression in late adulthood, and particularly apparent for those spending their childhoods in suburban settings. The association is robust to the inclusion of a host of household characteristics associated with childhood urbanicity, and is independent of contemporaneous urbanicity and contemporaneous income. When broken down by gender, we find some evidence of associations between depressive outcomes and urban living for men, and stronger evidence of such associations with urban and suburban living for women.

Conclusions: Our analysis reveals a relationship between childhood urbanicity and depression in late-adulthood. The evidence presented on the nature of this relationship is not straightforward but broadly suggestive of a link, differing by gender, between greater urbanicity and higher levels of depressive symptoms. The life-long nature of this association may potentially inform policy agendas aimed at improving urban & suburban living conditions.

Strengths & limitations of this study

- This is the first paper, to the best of our knowledge, to focus on long-arm relationships between childhood urbanicity of residence and late-adulthood depressive outcomes specifically.
- Due to the administrative structure of our data and the absence of any plausible identifying restrictions for causal effects, we are unable to make strong claims regarding causality.

Keywords: urbanisation; depression; mental health; lifecourse / childhood circumstances; ageing.

For peer review only - http://bmjopen.bmj.com/site/about/guidelines.xhtml

INTRODUCTION

The increase in prevalence of mental health disorders in the late 20th and early 21st century, along with increased urban dwelling, raises the question of interplay between these two phenomena. While a substantial body of literature, outlined below, examines links between the lived environment and mental health outcomes at various points over the life course, a potential long-arm relationship between childhood urbanicity and late-life depression remains less substantially researched.

A World Health Organisation review[1] reports that an estimated 300 million people suffer from depression globally. Associations with further health conditions and wider adverse socioeconomic outcomes are well-explored, with one meta-analysis[2] evidencing the multiplicity of social costs of major depression, such as reduced educational outcomes, adverse labour market and health outcomes, and increases in other health-damaging behaviours.

As of 2014, 54% of the world's population reside in urban areas, with this figure having risen from 30% since 1950 and being set to rise to 66% by 2050[3]. Various pathways have been suggested for links between levels of urbanicity and health outcomes. Vlahov & Galea [4] report that the urban social environment can affect individuals' general health via certain features such as socioeconomic status, crime and violence, presence of marginalised populations and the prevalence of psychological stressors. Diez Roux & Mair[5] further detail potential pathways for links between physical and social aspects of the lived environment and health outcomes. Compared to living in a rural area, urban dwellers face an elevated amount of environmental stressors on a daily basis which can lead to long-term degradation of their mental well-being. A further Finnish study[6] suggests that this may be due in part to variation in socioeconomic status between rural and urban areas.

For peer review only - http://bmjopen.bmj.com/site/about/guidelines.xhtml

Existing literature has generally focused on the relationships between urbanicity and contemporaneous health outcomes at various stages of the life course, with a number of these covering common mental disorders such as depression[7–12]. While the authors caution against drawing causal conclusions, in a broad meta-analysis of the link between urbanicity and mental health disorders, Penkalla & Kohler[13] find links between the degree of urbanicity and mood and anxiety disorders, psychosis, and substance use disorders. The largest single study[14], using administrative data collected on the entire Swedish population of 4.4 million people aged 25-64, finds that those in the top quintile by urbanicity have a 68-77% higher risk of developing psychosis and 12-20% higher risk of developing depression than those in the lowest, with these relationships being stronger for women than men. Oliver[15] discusses potential links between contemporary suburban and urban living and adverse mental health outcomes.

The relationship between childhood urbanicity and depression later in life remains a topic substantially less explored in existing research. Indeed, we believe there to exist only one study[16] to attempt to carry out preliminary investigations into longer-term and later-life relationships. Literature on the life course perspective[17–20] suggests, however, theoretical roles for the impact of conditions throughout an individual's life (in particular of early life conditions) on subsequent health, with this impact likely to vary in particular by gender (as evidenced by theoretical and empirical reviews[21,22]). Various pathways have been proposed to explain the incidence of depression specifically over the lifecycle, with Rudenstine[23] emphasising a role for the impact of early life circumstances.

Previous studies examining longer-arm relationships between urbanicity and mental ill-health suggest empirical support for these theoretical pathways. One study employing the records of over 2.3 million people born in Sweden between 1967 and 1989[24], finds that the population density of area of residence at age 15 is predictive of subsequent schizophrenia. Kim, Stewart,

For peer review only - http://bmjopen.bmj.com/site/about/guidelines.xhtml

Shin, Yoon, & Lee[16] use the principal areas that participants lived in during early-life (up to age 20) and mid-life (21-60) to investigate the relationship between the urbanicity of residence at such ages and depression later in life. The study finds links between current urbanicity and current depressive outcomes, and a greater prevalence of depression among women. Individuals who lived their entire life in rural areas are found to have the lowest rates of depression, with similar rates of depression observed among individuals who grew up in rural areas and subsequently lived in urban areas, and those who spent their entire life in urban areas. A further study[25] uses German and Chinese data to investigate links between childhood urbanicity and emotional characteristics later in (mostly young) adulthood. These results, drawn from relatively small samples (324 individuals in Germany, 713 in China) provide some evidence suggestive of a relationship with negative emotional traits for Chinese women alone, among whom *lower* symptoms of "fear" are exhibited for those who lived in urban settings in childhood.

In this paper, we seek to investigate specifically the relationship between early-life urban living and the presence of depression in late adulthood. We employ a cross-country European dataset of individuals aged 50 and above in order to investigate such a relationship, using individuals' self-reports of childhood circumstances, and depressive symptoms as measured by the EURO-D questionnaire. Section 2 introduces our data source and variables used in our study. Section 3 discusses the results of our study and its implications. Section 4 concludes.

For peer review only - http://bmjopen.bmj.com/site/about/guidelines.xhtml

METHODS

Data and sample

For the purpose of our study we employ two waves of the Survey of Health, Ageing and Retirement in Europe (SHARE), a pan-European survey containing information on health, socio-economic and social and family networks of individuals that are aged 50 or older in thirteen European countries plus Israel[1]. SHARE consists of six published waves to date, with individuals tracked across successive surveys. The study design and sampling methods, both in general and for the specific waves here employed, are detailed by the study's authors in several publications[26–30]. We employ the second contemporaneous wave of the study and SHARELIFE, the third wave of the survey, which focuses on the self-reported life histories of respondents. In total, 25,341 individuals appear in both datasets. After listwise removal of individuals with missing data for individual variables, our final sample stands at 20,400 (9029 men and 11,371 women) individuals[2], and 13,548 (6,154 men and 7,394 women) when control is made for contemporary urbanicity. A summary of item missingness is shown in Table A3. Table 1 shows the summary statistics for the final sample used in the study, broken down by gender and by childhood urbanicity.

Variables

The main dependent variable used in this study is depression, with symptoms measured by the EURO-D scale included in the SHARE questionnaire. The EURO-D is a symptoms scale for depression and has been validated in a cross-European study[31]. The scale covers 12 symptoms: depressed mood, pessimism, suicidality, guilt, sleep, interest, irritability, appetite, fatigue, concentration, enjoyment and tearfulness. The final EURO-D score (between 0 and

[1]Israel is, however, not included in our analysis due to its absence from wave 3.
[2]We also use a larger sample (consisting of 24,854 individuals) for our full information maximum likelihood analysis, containing all individuals in both waves of the data with a recorded EURO-D score.

For peer review only - http://bmjopen.bmj.com/site/about/guidelines.xhtml

12) is calculated from the total number of these symptoms present, with a score of 4 or more found to be an optimal cut-off point for the prediction of depression[31,32]. In addition to a set of models which treat this EURO-D score as the dependent variable in OLS regressions, in a second set of models, we transform this variable into a dichotomous variable that is equal to 1 for EURO-D scores 4 and higher, and 0 for scores strictly below 4.

The main independent variable of interest in our study is the urbanicity of the respondents' childhood homes. In the retrospective SHARELIFE questionnaire, respondents provided information on each dwelling they had lived in since birth, providing the exact year in which they moved, and the type of area in which each dwelling was located. This area type is coded in SHARELIFE with values of 1 ("A big city"), 2 ("The suburbs or outskirts of a big city"), 3 ("A large town"), 4 ("A small town"), 5 ("A rural area or village").

Durations, measured in years, between moving into and out of each dwelling were computed to determine the length that respondents lived in each of these. We then identified the home that respondents lived in for the longest time between birth and the age of 15, and the corresponding area type of that dwelling. In our base case, we include this variable as is, after cleaning.

Model specifications

We estimate our models primarily as a series of ordinary least squares (OLS) and logistic regressions – the former being used for regressions using the EURO-D score as dependent variable and the latter for those using a dichotomised EURO-D variable equal to 1 for scores of 4 or more, and to 0 for scores strictly fewer than 4. We also include a full information maximum likelihood (FIML) specification, which allows us to estimate parameters using all available data in the sample under the assumption that observations are missing at random[33,34], as a final robustness check.

For peer review only - http://bmjopen.bmj.com/site/about/guidelines.xhtml

We include basic demographic controls of gender and age-period-cohort in all models, as well as allowing for country fixed effects. In summarising the varying social, marital and economic positions of men and women and the potential tendencies of each to respond to adverse life events in different ways, existing literature[21,35] suggests there may be differences both in overall prevalence of depression by gender, and also in relationships between explanatory factors and such mental health outcomes. Furthermore, while all respondents in the SHARELIFE survey are aged above 50, existing literature points to a role for age as a relevant control variable even among this group[36,37]. Although interpretation of the variation explained by age variables in studies such as this is plagued by problems in separately identifying age-period-cohort effects – that is, due to collinearity, identifying these three effects independently is impossible without resorting to additional, untestable, assumptions[38] – we include these variables purely as controls for the combination of all three potential such effects. In an initial parsimonious model, we control only for these basic demographic factors.

In further models, we also include additional control variables related to childhood circumstances and characteristics, and contemporaneous log income[3]. Latham[41] reports that childhood disability is significantly associated with later-life depression, a relationship found to be of an indirect nature through social and health factors such as midlife physical health. To control for such an effect in our model, we introduce self-reported childhood health status, a score between 1 (poor) to 5 (excellent). We also seek to control for childhood socioeconomic circumstances and, to this end, follow a validated method[42] employed in other papers[43,44] employing SHARE, a principal components score, created from a combination of measures of childhood (age 10) socioeconomic status included in SHARELIFE. Table A1 in the Appendix provides the results of our principal component analysis. We use only component 1 to construct

[3]We implement this by dividing gross household income by the square root of household size, as per the current method employed by recent OECD publications[39,40].

For peer review only - http://bmjopen.bmj.com/site/about/guidelines.xhtml

the score used in our index of childhood SES, which we divide into deciles in our model. We also add parental presence (split by step- and biological parent) in the childhood home at the age of ten, self-perceived and self-recalled ability at age ten in mathematics and literacy compared to the average and self-reported parental alcohol abuse.

In a final model, we seek to consider the relationship between depression in late adulthood and childhood urbanicity once control is made for contemporaneous urbanicity at the time at which the SHARE interview was conducted. To this end, we include a variable for current urbanicity coded in an identical way to that in which we code childhood urbanicity, as earlier described.

We also present alternative specifications, changing the dependent variable to the urbanicity of area of residence when aged between 0-5, 6-10, and 11-15 years.

For peer review only - http://bmjopen.bmj.com/site/about/guidelines.xhtml

RESULTS

[Table 1 here]

Out of the 20,400 individuals included in our main sample, 23% recorded a EURO-D score of 4 or above, with an average score of 2.18. We find that women (29%) were almost twice as likely as men (15%) to exhibit a EURO-D score of 4 or above, and this score for women (2.58) was just under one point higher on average than for men (1.68).

[Tables 2-5 here]

Our results suggest that the urbanicity of childhood residence, rather than of current residence, is of primary importance in the relationship between the lived environment and depression. They also suggest that such a relationship varies by gender, with an F-test of the interactions between gender and childhood urbanicity categories being rejected at $p < 0.0001$. A matrix of individuals' urbanicity of residence in childhood and currently is presented in Table A2.

Our results suggest a complex relationship between level of childhood urbanicity and later life depression. A parsimonious pooled regression (Column 1, Table 2) including both men and women suggests that, compared to individuals living in rural areas or villages, individuals spending their childhoods in cities or suburbs of big cities are observed to have significantly worse outcomes in late adulthood. Our model with childhood controls (Column 2, Table 2) suggests that individuals growing up in big cities are observed to have EURO-D scores of 0.0978 ($p < 0.05$) on average worse than those growing up in rural areas or villages. In the same model, individuals growing up in the suburbs of big cities have worse still depressive outcomes as measured by the EURO-D score, with this score being on average 0.153 ($p < 0.01$)

For peer review only - http://bmjopen.bmj.com/site/about/guidelines.xhtml

higher for such individuals. Individuals growing up in small towns are estimated to have a EURO-D score in adulthood on average 0.0760 ($p < 0.10$) higher than those growing up in rural areas.

When broken down by gender, these relationships across our models appear to differ substantially. Much stronger results are observed for women than for men. While only the coefficient for having spent childhood in a big city reaches conventional levels of significance for men (Column 4, Table 2), this relationship is only observed for women for those who grew up in the suburbs of a big city (Column 6, Table 2). Men with childhoods spent in a big city are estimated to have a EURO-D score of on average 0.104 ($p < 0.10$) higher than those who grew up in rural areas or villages, while women with suburban childhoods are observed to have a EURO-D score of on average 0.223 ($p < 0.01$) higher than those who grew up in rural areas or villages.

While these may appear to represent small increases, it is instructive to also consider the results of a logit model with presence of a EURO-D score of 4 or more as dependent variable, with these results presented in Tables 3 (odds-ratios) and 4 (average marginal effects). Such a model again suggests that suburban childhoods are associated with worse depressive outcomes for women, with an average marginal effect estimated for women of 5.58 percentage points in our preferred model.

While we confirm a role for childhood urbanicity in explaining adult urbanicity (with an F-test of the relevance of such variables in an ordered logit model with adulthood urbanicity as the dependent variable being rejected at $p < 0.0001$), the addition of this as an explanatory variable (Columns 3, 5 & 7, Table 2) does not substantially affect our estimated relationship between urbanicity of childhood residence and the individual's EURO-D score in late adulthood.

For peer review only - http://bmjopen.bmj.com/site/about/guidelines.xhtml

DISCUSSION

These results are suggestive of a long arm in the relationship between childhood urbanicity and mental health conditions which still persists after beyond the age of 50. Our results are robust to several model specifications.

Our findings can also be viewed as a positive relationship between urbanicity and the presence of mental health conditions, with this relationship driven primarily by a stronger and more statistically significant relationship among women. Contra existing literature, we find that increasing levels of contemporaneous urbanicity generally have a small and non-significant negative relationship with both EURO-D score (in our linear regressions) and presence of a EURO-D score of 4 or more (in our logistic regressions). Furthermore, the inclusion of this additional variable does not substantially impact upon the magnitude of our parameter estimates of the relationship between childhood urbanicity and depressive outcomes. These small and statistically insignificant parameter estimates suggest little evidence of a relationship between current urbanicity and the presence of depression in our sample.

With the continued trend of rapid urbanisation and the rising prominence of mental health conditions[1,3], the investigation of links between childhood urbanicity and mental health outcomes is required to help policy makers better understand and design policies to cater to such growing needs. Our study follows in the footsteps of existing literature on urbanicity and mental health but goes on to specifically look at the relationship of urbanicity and depression from a long-term standpoint, as well as being, to our knowledge, the first of this type to consider separate relationships by gender. In contrast to existing studies in the field, our study employs a cross-national sample with a comprehensive and wide range of retrospective data.

In line with literature in the life course perspective emphasising the importance of early life circumstances, we find that spending one's childhood in an urban or suburban area has a

For peer review only - http://bmjopen.bmj.com/site/about/guidelines.xhtml

positive and significant relationship with the presence of depression in later-life adulthood when a pooled regression is run for the entire sample. This relationship persists even after controlling for other socioeconomic, demographic and environmental factors. This relationship appears to be particularly strong for women. Individuals that spend the longest time in their childhood in a suburban home face an average increase in probability of 3.4 percentage points (5.58 points for women) of reporting four or more depressive symptoms on the EURO-D scale during later-life adulthood, a relationship that persists even when control is made for contemporaneous urbanicity. While we are unable to establish any identifying restrictions for causal effects in our dataset, the findings here are striking and establish potential pathways for future research. Although SHARE's rich retrospective information, even if based on recall data, provides a rare opportunity to investigate long-arm relationships such as this, the use of linked administrative long panel datasets would be preferable. Such datasets would contain more comprehensive information about specific areas of residence and would help to overcome one limitation of our study: that of treating "big city" as one homogeneous item, borne out of our inability to account for the fact that the experience of city life varies both within and between cities. Future research could seek to overcome some of these inherent limitations of our approach, in seeking to establish causal effects, as well as to establish more clearly theoretical pathways for such effects.

For peer review only - http://bmjopen.bmj.com/site/about/guidelines.xhtml

APPENDIX

[Tables A1-A4 here]

For peer review only

For peer review only - http://bmjopen.bmj.com/site/about/guidelines.xhtml

References

1 World Health Organisation. Mental disorders. WHO. 2017.http://www.who.int/mediacentre/factsheets/fs396/en/ (accessed 7 Aug 2017).

2 Kessler RC. The Costs of Depression. *Psychiatr Clin* 2012;**35**:1–14. doi:10.1016/j.psc.2011.11.005

3 United Nations, Department of Economic and Social Affairs, Population Division. *World Urbanization Prospects, the 2014 Revision: Highlights*. 2014.

4 Vlahov D, Galea S. Urbanization, urbanicity, and health. *J Urban Health Bull N Y Acad Med* 2002;**79**:S1–12. doi:10.1093/jurban/79.suppl_1.S1

5 Diez Roux AV, Mair C. Neighborhoods and health. *Ann N Y Acad Sci* 2010;**1186**:125–45. doi:10.1111/j.1749-6632.2009.05333.x

6 Airaksinen J, Hakulinen C, Elovainio M, *et al.* Neighborhood effects in depressive symptoms, social support, and mistrust: Longitudinal analysis with repeated measurements. *Soc Sci Med* 2015;**136**–**137**:10–6. doi:10.1016/j.socscimed.2015.04.034

7 Kim M-H, Jung-Choi K, Jun H-J, *et al.* Socioeconomic inequalities in suicidal ideation, parasuicides, and completed suicides in South Korea. *Soc Sci Med* 2010;**70**:1254–61. doi:10.1016/j.socscimed.2010.01.004

8 Maas J, Verheij RA, Groenewegen PP, *et al.* Green space, urbanity, and health: how strong is the relation? *J Epidemiol Community Health* 2006;**60**:587–92. doi:10.1136/jech.2005.043125

9 Newbury J, Arseneault L, Caspi A, *et al.* Why Are Children in Urban Neighborhoods at Increased Risk for Psychotic Symptoms? Findings From a UK Longitudinal Cohort Study. *Schizophr Bull* 2016;**42**:1372–83. doi:10.1093/schbul/sbw052

10 van de Poel E, O'Donnell O, van Doorslaer E. Urbanization and the spread of diseases of affluence in China. *Econ Hum Biol* 2009;**7**:200–16. doi:10.1016/j.ehb.2009.05.004

11 Weich S, Twigg L, Holt G, *et al.* Contextual risk factors for the common mental disorders in Britain: a multilevel investigation of the effects of place. *J Epidemiol Community Health* 2003;**57**:616–21. doi:10.1136/jech.57.8.616

12 Kovess-Masféty V, Alonso J, de Graaf R, *et al.* A European approach to rural-urban differences in mental health: the ESEMeD 2000 comparative study. *Can J Psychiatry Rev Can Psychiatr* 2005;**50**:926–36. doi:10.1177/070674370505001407

13 Penkalla AM, Kohler S. Urbanicity and Mental Health in Europe: A Systematic Review. *Eur J Ment Health* 2014;**9**:163–77.

14 Sundquist K, Frank G, Sundquist J. Urbanisation and incidence of psychosis and depression: follow-up study of 4.4 million women and men in Sweden. *Br J Psychiatry J Ment Sci* 2004;**184**:293–8.

For peer review only - http://bmjopen.bmj.com/site/about/guidelines.xhtml

15 Oliver JE. Mental Life and the Metropolis in Suburban America: The Psychological Correlates of Metropolitan Place Characteristics. *Urban Aff Rev* 2003;**39**:228–53. doi:10.1177/1078087403254445

16 Kim J-M, Stewart R, Shin I-S, *et al.* Lifetime urban/rural residence, social support and late-life depression in Korea. *Int J Geriatr Psychiatry* 2004;**19**:843–51. doi:10.1002/gps.1175

17 Ben-Shlomo Y, Kuh D. A life course approach to chronic disease epidemiology: conceptual models, empirical challenges and interdisciplinary perspectives. *Int J Epidemiol* 2002;**31**:285–93. doi:10.1093/ije/31.2.285

18 Koenen KC, Rudenstine S, Susser E, *et al.*, editors. *A Life Course Approach to Mental Disorders*. Oxford, New York: : Oxford University Press 2013.

19 Kuh D, Ben Shlomo Y, editors. *A Life Course Approach to Chronic Disease Epidemiology*. Second Edition. Oxford, New York: : Oxford University Press 2004.

20 Pudrovska T, Anikputa B. Early-life socioeconomic status and mortality in later life: an integration of four life-course mechanisms. *J Gerontol B Psychol Sci Soc Sci* 2014;**69**:451–60. doi:10.1093/geronb/gbt122

21 Cleary PD. Gender differences in stress-related disorders. In: Barnett RC, Biener L, Baruch GK, eds. *Gender and stress*. New York, NY, US: : Free Press 1987. 39–72.

22 Van de Velde S, Bracke P, Levecque K. Gender differences in depression in 23 European countries. Cross-national variation in the gender gap in depression. *Soc Sci Med 1982* 2010;**71**:305–13. doi:10.1016/j.socscimed.2010.03.035

23 Rudenstine S. Applying a life course perspective to depression. In: Koenen KC, Rudenstine S, Susser E, *et al.*, eds. *A Life Course Approach to Mental Disorders*. Oxford University Press 2013. 88–96. doi:10.1093/acprof:oso/9780199657018.003.0008

24 Sariaslan A, Larsson H, D'Onofrio B, *et al.* Does Population Density and Neighborhood Deprivation Predict Schizophrenia? A Nationwide Swedish Family-Based Study of 2.4 Million Individuals. *Schizophr Bull* 2015;**41**:494–502. doi:10.1093/schbul/sbu105

25 Sindermann C, Kendrick KM, Becker B, *et al.* Does Growing up in Urban Compared to Rural Areas Shape Primary Emotional Traits? *Behav Sci Basel Switz* 2017;**7**. doi:10.3390/bs7030060

26 Börsch-Supan A. Survey of Health, Ageing and Retirement in Europe (SHARE) Wave 2. 2017. doi:10.6103/SHARE.w2.600

27 Börsch-Supan A. Survey of Health, Ageing and Retirement in Europe (SHARE) Wave 3 - SHARELIFE. 2017. doi:10.6103/SHARE.w3.600

28 Börsch-Supan A, Brugiavini A, Jürges H, *et al. First Results from the Survey of Health, Ageing, and Retirement in Europe (2004-2007) : Starting the longitudinal dimension*. Mannheim: : Mannheim Research Institute for the Economics of Aging 2008. http://www.share-

For peer review only

For peer review only - http://bmjopen.bmj.com/site/about/guidelines.xhtml

project.org/fileadmin/pdf_documentation/FRB2/FRB2_all_chapters.pdf (accessed 11 Apr 2017).

29 Börsch-Supan A, Brandt M, Hunkler C, *et al.* Data Resource Profile: the Survey of Health, Ageing and Retirement in Europe (SHARE). *Int J Epidemiol* 2013;**42**:992–1001. doi:10.1093/ije/dyt088

30 Börsch-Supan A, Schröder M. Retrospective data collection in the Survey of Health, Ageing and Retirement in Europe. *SHARELIFE Methodol* 2011;**5**.http://www.share-project.org/uploads/tx_sharepublications/FRB-Methodology_feb2011_color-1.pdf#page=6

31 Prince MJ, Reischies F, Beekman AT, *et al.* Development of the EURO-D scale--a European, Union initiative to compare symptoms of depression in 14 European centres. *Br J Psychiatry* 1999;**174**:330–8. doi:10.1192/bjp.174.4.330

32 Prince MJ, Beekman AT, Deeg DJ, *et al.* Depression symptoms in late life assessed using the EURO-D scale. Effect of age, gender and marital status in 14 European centres. *Br J Psychiatry J Ment Sci* 1999;**174**:339–45.

33 Schafer JL, Graham JW. Missing data: Our view of the state of the art. *Psychol Methods* 2002;**7**:147–77. doi:10.1037/1082-989X.7.2.147

34 Enders CK. The Performance of the Full Information Maximum Likelihood Estimator in Multiple Regression Models with Missing Data. *Educ Psychol Meas* 2001;**61**:713–40. doi:10.1177/0013164401615001

35 Simon RW. Revisiting the relationships among gender, marital status, and mental health. *AJS* 2002;**107**:1065–96.

36 Keyes M, Legrand LN, Iacono WG, *et al.* Parental Smoking and Adolescent Problem Behavior: An Adoption Study of General and Specific Effects. *Am J Psychiatry* 2008;**165**:1338–44. doi:10.1176/appi.ajp.2008.08010125

37 Mirowsky J, Ross CE. Age and Depression. *J Health Soc Behav* 1992;**33**:187–205. doi:10.2307/2137349

38 Bell A, Jones K. The impossibility of separating age, period and cohort effects. *Soc Sci Med 1982* 2013;**93**:163–5. doi:10.1016/j.socscimed.2013.04.029

39 Organisation for Economic Co-operation and Development. Divided We Stand: Why Inequality Keeps Rising: An overview of growing income inequalities in OECD countries: main findings. *OECD Publ* Published Online First: 2011.http://www.ingentaconnect.com/content/oecd/16815378/2011/00002011/00000014/8111111ec003

40 Organisation for Economic Co-operation and Development. What are equivalence scales? *OECD Proj Income Distrib Poverty* 2013.

41 Latham K. The 'long arm' of childhood health: linking childhood disability to late midlife mental health. *Res Aging* 2015;**37**:82–102. doi:10.1177/0164027514522276

For peer review only - http://bmjopen.bmj.com/site/about/guidelines.xhtml

42 Havari E, Mazzonna F. Can We Trust Older People's Statements on Their Childhood Circumstances? Evidence from SHARELIFE. *Eur J Popul* 2015;**31**:233–57. doi:10.1007/s10680-014-9332-y

43 Angelini V, Mierau J. Late-life health effects of teenage motherhood. *Demogr Res* 2018;**39**:1081–104. doi:10.4054/DemRes.2018.39.41

44 Angelini V, Howdon DDD, Mierau JO. Childhood Socioeconomic Status and Late-Adulthood Mental Health: Results from the Survey on Health, Ageing and Retirement in Europe. *J Gerontol B Psychol Sci Soc Sci* Published Online First: 16 March 2018. doi:10.1093/geronb/gby028

For peer review only

Tables

TABLE 1
Descriptive Statistics (N = 20,400)

Name	Mean All	St. Dev. All	Mean Men	Mean Women	
**Individual Characteristics**			(N=9,029)	(N=11,371)	
Male	0.44	0.50	N/A	N/A	
Age	64.23	9.43	64.34	64.15	
Urbanicity of current residence	3.33	1.41	3.35	3.31	
Urbanicity of childhood residence	3.70	1.46	3.67	3.71	
Equivalised HH income (000s)	21.57	30.69	23.36	20.16	***
**Health Status**					
Depression (EURO-D)	2.18	2.15	1.68	2.58	***
Depression (0/1)	0.23	0.42	0.15	0.29	***
**Childhood SES**					
Number of Rooms	3.72	1.79	3.73	3.71	
Number of Facilities	2.28	1.48	2.28	2.28	
Number of Books (1 = few to 5 = >200 books)	2.13	1.21	2.11	2.14	
Occupation of Main Breadwinner (ranked)	2.90	0.80	2.90	2.91	
**Childhood Characteristics**					
Math Performance (1 = low to 5 = high)	3.30	0.89	3.39	3.23	***
Language Performance (1 = low to 5 = high)	3.33	0.87	3.24	3.41	***
Self-Reported Health (1 = poor to 5 = excellent)	3.97	1.00	4.04	3.91	***
Parent heavy drinker	0.08	0.27	0.08	0.08	
Biological Father Present	0.91	0.28	0.91	0.91	
Biological Mother Present	0.96	0.19	0.97	0.96	*
Stepfather Present	0.02	0.15	0.02	0.02	
Stepmother Present	0.02	0.12	0.01	0.02	

Note: */**/*** indicate significant differences at the 10%/5%/1% level based on Student's t-tests of group mean comparisons.

For peer review only

TABLE 2

Association Between Childhood Urbanicity and Late-Adulthood EURO-D score (OLS coefficients)

		Full Sample		Men	Men	Women	Women
Dependent variable	EURO-D	EURO-D	EURO-D	EURO-D	EURO-D	EURO-D	EURO-D
	(1)	(2)	(3)	(4)	(5)	(6)	(7)
Age 0-15 living environment (omitted: rural area or village)							
Big city	0.0474	0.0978**	0.117**	0.104*	0.0755	0.0937	0.151*
	(0.0432)	(0.0440)	(0.0566)	(0.0582)	(0.0740)	(0.0641)	(0.0836)
Suburbs or outskirts of a big city	0.117**	0.153***	0.156**	0.0563	0.0355	0.223***	0.253**
	(0.0560)	(0.0562)	(0.0704)	(0.0751)	(0.0913)	(0.0812)	(0.105)
Large town	0.0208	0.0760*	0.0977*	0.0653	0.0812	0.0834	0.124
	(0.0433)	(0.0436)	(0.0558)	(0.0583)	(0.0726)	(0.0631)	(0.0826)
Small town	-0.0275	0.0136	0.0153	0.0818	0.114*	-0.0471	-0.0791
	(0.0412)	(0.0410)	(0.0518)	(0.0552)	(0.0683)	(0.0590)	(0.0759)
Male	-0.887***	-0.846***	-0.864***				
	(0.0286)	(0.0287)	(0.0355)				
Current living environment (omitted: rural area or village)							
Big city			-0.0850		-0.0755		-0.0634
			(0.0603)		(0.0800)		(0.0883)
Suburbs or outskirts of a big city			-0.0528		0.0267		-0.115
			(0.0588)		(0.0759)		(0.0879)
Large town			0.0299		-0.0670		0.136*
			(0.0541)		(0.0715)		(0.0792)
Small town			-0.0840		-0.0868		-0.0669
			(0.0514)		(0.0671)		(0.0760)

For peer review only

For peer review only - http://bmjopen.bmj.com/site/about/guidelines.xhtml

Additional childhood controls & contemporaneous income	NO	YES	YES	YES	YES	YES	YES
Observations	20,400	20,400	13,548	9,029	6,154	11,371	7,394

*Notes: Constant not reported. */**/*** indicate statistical significance at the 10%/5%/1% level, standard errors between brackets.*

All models contain controls for age-period-cohort and country fixed effects.

For peer review only

For peer review only - http://bmjopen.bmj.com/site/about/guidelines.xhtml

TABLE 3
Association Between Childhood Urbanicity and Late-Adulthood Depression incidence (Logit, odds ratios)

	Full Sample			Men	Men	Women	Women
	(1)	(2)	(3)	(4)	(5)	(6)	(7)
Age 0-15 living environment (omitted: rural area or village)							
Big city	1.045	1.121**	1.100	1.129	1.021	1.117	1.147
	(0.0566)	(0.0634)	(0.0797)	(0.109)	(0.127)	(0.0783)	(0.103)
Suburbs or outskirts of a big city	1.176**	1.235***	1.217**	1.039	0.990	1.334***	1.350***
	(0.0810)	(0.0876)	(0.108)	(0.132)	(0.152)	(0.115)	(0.148)
Large town	1.036	1.109*	1.087	1.084	1.047	1.120*	1.116
	(0.0556)	(0.0615)	(0.0765)	(0.105)	(0.127)	(0.0761)	(0.0976)
Small town	0.996	1.045	1.057	1.106	1.143	1.014	1.007
	(0.0504)	(0.0540)	(0.0685)	(0.0993)	(0.125)	(0.0643)	(0.0812)
Male	0.399***	0.410***	0.416***				
	(0.0149)	(0.0157)	(0.0193)				
Current living environment (omitted: rural area or village)							
Big city			0.947		0.927		0.980
			(0.0716)		(0.121)		(0.0916)
Suburbs or outskirts of a big city			0.984		1.071		0.945
			(0.0737)		(0.132)		(0.0890)
Large town			1.052		0.915		1.155*
			(0.0705)		(0.106)		(0.0961)
Small town			0.871**		0.825*		0.906
			(0.0565)		(0.0908)		(0.0732)
Additional childhood controls & contemporaneous income	NO	YES	YES	YES	YES	YES	YES
Observations	20,400	20,400	13,548	9,029	6,154	11,371	7,394

For peer review only - http://bmjopen.bmj.com/site/about/guidelines.xhtml

*Notes: Constant not reported. */**/*** indicate statistical significance at the 10%/5%/1% level, standard errors between brackets.*

All models contain controls for age-period-cohort and country fixed effects.

For peer review only

For peer review only - http://bmjopen.bmj.com/site/about/guidelines.xhtml

TABLE 4

Association Between Childhood Urbanicity and Late-Adulthood Depression incidence (Logit, average marginal effects)

	Full Sample			Men	Men	Women	Women
	(1)	(2)	(3)	(4)	(5)	(6)	(7)
Age 0-15 living environment (omitted: rural area or village)							
Big city	0.00717	0.0181**	0.0151	0.0144	0.00240	0.0208	0.0261
	(0.00878)	(0.00903)	(0.0116)	(0.0117)	(0.0147)	(0.0133)	(0.0173)
Suburbs or outskirts of a big city	0.0268**	0.0340***	0.0319**	0.00443	-0.00118	0.0558***	0.0586***
	(0.0117)	(0.0118)	(0.0147)	(0.0148)	(0.0179)	(0.0172)	(0.0220)
Large town	0.00569	0.0162*	0.0132	0.00944	0.00546	0.0212*	0.0209
	(0.00868)	(0.00881)	(0.0112)	(0.0115)	(0.0144)	(0.0129)	(0.0167)
Small town	-0.000625	0.00683	0.00875	0.0118	0.0163	0.00253	0.00129
	(0.00806)	(0.00806)	(0.0102)	(0.0107)	(0.0136)	(0.0117)	(0.0150)
Male	-0.148***	-0.141***	-0.140***				
	(0.00580)	(0.00583)	(0.00714)				
Current living environment (omitted: rural area or village)							
Big city			-0.00870		-0.00923		-0.00388
			(0.0120)		(0.0157)		(0.0178)
Suburbs or outskirts of a big city			-0.00255		0.00866		-0.0108
			(0.0120)		(0.0157)		(0.0178)
Large town			0.00823		-0.0107		0.0282*
			(0.0109)		(0.0139)		(0.0163)
Small town			-0.0216**		-0.0225*		-0.0185
			(0.0101)		(0.0128)		(0.0151)

For peer review only - http://bmjopen.bmj.com/site/about/guidelines.xhtml

Additional childhood controls & contemporaneous income	NO	YES	YES	YES	YES	YES	YES
Observations	20,400	20,400	13,548	9,029	6,154	11,371	7,394

*Notes: Constant not reported. */**/*** indicate statistical significance at the 10%/5%/1% level, standard errors between brackets.*

All models contain controls for age-period-cohort and country fixed effects.

TABLE 4

Association Between Childhood Urbanicity and Late-Adulthood Depression incidence (Logit, average marginal effects)

	Full Sample			Men	Men	Women	Women
	(1)	(2)	(3)	(4)	(5)	(6)	(7)
Age 0-15 living environment (omitted: rural area or village)							
Big city	0.00717	0.0181**	0.0151	0.0144	0.00240	0.0208	0.0261
	(0.00878)	(0.00903)	(0.0116)	(0.0117)	(0.0147)	(0.0133)	(0.0173)
Suburbs or outskirts of a big city	0.0268**	0.0340***	0.0319**	0.00443	-0.00118	0.0558***	0.0586***
	(0.0117)	(0.0118)	(0.0147)	(0.0148)	(0.0179)	(0.0172)	(0.0220)
Large town	0.00569	0.0162*	0.0132	0.00944	0.00546	0.0212*	0.0209
	(0.00868)	(0.00881)	(0.0112)	(0.0115)	(0.0144)	(0.0129)	(0.0167)
Small town	-0.000625	0.00683	0.00875	0.0118	0.0163	0.00253	0.00129
	(0.00806)	(0.00806)	(0.0102)	(0.0107)	(0.0136)	(0.0117)	(0.0150)
Male	-0.148***	-0.141***	-0.140***				
	(0.00580)	(0.00583)	(0.00714)				
Current living environment (omitted: rural area or village)							
Big city			-0.00870		-0.00923		-0.00388

For peer review only - http://bmjopen.bmj.com/site/about/guidelines.xhtml

			(0.0120)		(0.0157)		(0.0178)
Suburbs or outskirts of a big city			-0.00255		0.00866		-0.0108
			(0.0120)		(0.0157)		(0.0178)
Large town			0.00823		-0.0107		0.0282*
			(0.0109)		(0.0139)		(0.0163)
Small town			-0.0216**		-0.0225*		-0.0185
			(0.0101)		(0.0128)		(0.0151)
Additional childhood controls & contemporaneous income	NO	YES	YES	YES	YES	YES	YES
Observations	20,400	20,400	13,548	9,029	6,154	11,371	7,394

*Notes: Constant not reported. */**/*** indicate statistical significance at the 10%/5%/1% level, standard errors between brackets.*

All models contain controls for age-period-cohort and country fixed effects.

For peer review only

TABLE A1
Principal component analysis for childhood SES

	Component			
	1st	2nd	3rd	4th
Number of Rooms	0.4073	0.8637	-0.2968	-0.0111
Number of Books (grouping)	0.5651	-0.1752	0.2367	0.7707
Occupation of Main Breadwinner (ranked)	-0.4758	0.4695	0.7042	0.2394
Number of Facilities	0.537	-0.0546	0.6	-0.5905

For peer review only

TABLE A2

Urbanicity of childhood residence and of current residence (all values percentages)

		Current residence					
		1	2	3	4	5	Total
Childhood residence	1	37.65	21.28	15.13	11.31	14.63	100
	2	17.19	39.64	14.03	14.84	14.30	100
	3	14.39	16.20	38.87	16.81	13.73	100
	4	11.14	12.62	20.40	38.10	17.75	100
	5	9.31	9.05	15.80	21.98	43.85	100
	Total	15.23	15.04	19.74	21.89	28.11	100

Key

1: A big city

2: The suburbs or outskirts of a big city

3: A large town

4: A small town

5: A rural area or village

For peer review only

For peer review only - http://bmjopen.bmj.com/site/about/guidelines.xhtml

Table A3
Item missingness: summary (all individuals in both waves, n=25,341)

Variable	Missing count	Present count
EURO-D	487	24,854
Childhood urban residence	1,715	23,626
Age	716	24,625
Childhood maths performance	859	24,482
Childhood language performance	927	24,414
Childhood self-reported health	189	25,152
Parent heavy drinker	103	25,238
Deprivation score	1,172	24,169
Biological Father Present	506	24,835
Biological Mother Present	506	24,835
Stepfather Present	506	24,835
Stepmother Present	506	24,835
Equivalised HH income	444	24,897
Current urban residence	8,882	16,459

For peer review only

For peer review only - http://bmjopen.bmj.com/site/about/guidelines.xhtml

TABLE A4

Association Between Stage of Childhood Urbanicity and Late-Adulthood Depression Outcomes

	OLS			Logit, average marginal effects		
	(1)	(2)	(3)	(4)	(5)	(6)
Age	0-5	6-10	11-15	0-5	6-10	11-15
Childhood living environment (omitted: rural area or village)						
Big city	0.0913**	0.104**	0.124***	0.0193**	0.0215**	0.0234***
	(0.0437)	(0.0440)	(0.0439)	(0.00889)	(0.00897)	(0.00893)
Suburbs or outskirts of a big city	0.173***	0.159***	0.194***	0.0234**	0.0209*	0.0152
	(0.0577)	(0.0562)	(0.0554)	(0.0117)	(0.0114)	(0.0114)
Large town	0.0706	0.0781*	0.0977**	0.00527	0.00831	0.00729
	(0.0439)	(0.0437)	(0.0434)	(0.00915)	(0.00912)	(0.00905)
Small town	-0.0119	0.0165	0.0187	-0.00171	0.00242	-0.00300
	(0.0410)	(0.0410)	(0.0409)	(0.00874)	(0.00874)	(0.00875)
Male	-0.845***	-0.845***	-0.845***	-0.121***	-0.121***	-0.121***
	(0.0287)	(0.0287)	(0.0287)	(0.00583)	(0.00583)	(0.00583)
Observations	20,400	20,400	20,400	20,400	20,400	20,400

*Notes: Constant not reported. */**/*** indicate statistical significance at the 10%/5%/1% level, standard errors between brackets.*

All models contain controls for age-period-cohort, country fixed effects and additional childhood controls & contemporaneous income.

For peer review only

BMJ Open

The Relationship Between Early Life Urbanicity and Depression in Late-Adulthood: Evidence from the Survey of Health, Ageing and Retirement in Europe

Journal:	*BMJ Open*
Manuscript ID	bmjopen-2018-028090.R1
Article Type:	Research
Date Submitted by the Author:	29-May-2019
Complete List of Authors:	Howdon, Daniel; University of Leeds Leeds Institute of Health Sciences, Academic Unit of Health Economics<br>Mierau, Jochen; Rijksuniversiteit Groningen Faculteit Economie en Bedrijfskunde, Economics, Econometrics and Finance<br>Liew, Samuel; Copenhagen Business School
**Primary Subject Heading**:	Public health
Secondary Subject Heading:	Health economics, Mental health
Keywords:	MENTAL HEALTH, HEALTH ECONOMICS, PUBLIC HEALTH

SCHOLARONE™
Manuscripts

The Relationship Between Early Life Urbanicity and Depression in Late-Adulthood: Evidence from the Survey of Health, Ageing and Retirement in Europe

Daniel Howdon *, 1, 2 Jochen O. Mierau 2, 3, 4 Samuel K. M. Liew

* Corresponding author. Academic Unit of Health Economics, Leeds Institute of Health Sciences, Level 10, Worsley Building, Clarendon Way, Leeds, LS2 9NL, United Kingdom. d.howdon@leeds.ac.uk

1: Academic Unit of Health Economics, Leeds Institute of Health Sciences, University of Leeds, UK

2: Department of Economics, Econometrics and Finance, Faculty of Economics and Business, University of Groningen, Groningen, Netherlands

3: NETSPAR, Tilburg, Netherlands

4: Aletta Jacobs School of Public Health, University of Groningen, Groningen, Netherlands.

Word count: 3509

For peer review only

Authorship statement:

SL carried out initial empirical estimation and wrote the first draft as a postgraduate thesis under the supervision of DH and JM. DH carried out re-estimation and estimation of additional model specifications, and substantially redrafted the paper. JM and SL made further redrafting contributions to the paper. All authors approved the final draft.

Data sharing:

The data is a panel dataset available from the SHARE website, with licensing subject to use in scientific research.

Quoting directly from the dataset's website:

http://www.share-project.org/data-access/user-registration.html

"SHARE-ERIC aims to share the SHARE data as soon as possible with the entire scientific community. Access to the SHARE data is provided free of charge on the basis of a release policy that gives quick and convenient access to all scientific users world-wide after individual registration. All details about the application and registration process can be found below.

SHARE data may be used for scientific research, subject to European Union and national data protection laws and the SHARE Conditions of Use. Furthermore, SHARE-ERIC promises to the participants in the study that the data will not be used for other purposes.

Therefore, any other uses, such as a commercial use of the data, are excluded as matter of principle.

In accordance with the legal requirements and ethical commitments related to the SHARE data collection, applicants have to acknowledge and agree that they will use the SHARE data only for scientific purposes. In this connection, they also have to provide relevant information with regard to their scientific alignment during the registration process."

For peer review only

Acknowledgements:

This paper uses data from SHARE Waves 2 and 3 (SHARELIFE) (DOIs: 10.6103/SHARE.w2.610, 10.6103/SHARE.w3.610), see Börsch-Supan et al. (2013) for methodological details. The SHARE data collection has been primarily funded by the European Commission through FP5 (QLK6-CT-2001-00360), FP6 (SHARE-I3: RII-CT-2006-062193, COMPARE: CIT5-CT-2005-028857, SHARELIFE: CIT4-CT-2006-028812) and FP7

For peer review only - http://bmjopen.bmj.com/site/about/guidelines.xhtml

(SHARE-PREP: N°211909, SHARE-LEAP: N°227822, SHARE M4: N°261982). Additional funding from the German Ministry of Education and Research, the Max Planck Society for the Advancement of Science, the U.S. National Institute on Aging (U01_AG09740-13S2, P01_AG005842, P01_AG08291, P30_AG12815, R21_AG025169, Y1-AG-4553-01, IAG_BSR06-11, OGHA_04-064, HHSN271201300071C) and from various national funding sources is gratefully acknowledged (see www.share-project.org).

We gratefully acknowledge the insightful comments of referees.

Ethics:

An ethics statement related to SHARE/SHARELIFE, the only data on which we rely for this paper, is available at http://www.share-project.org/fileadmin/pdf_documentation/SHARE_ethics_approvals.pdf.

Competing interests:

None to declare

Funding: This project was supported by the Investeringsagenda of the University of Groningen.

Patient and Public Involvement: no patient involvement.

For peer review only

Abstract

Objectives: We aim to study the association of childhood urbanicity with depressive symptoms in late adulthood.

Design, setting & participants: We use linear and logistic regression to analyse data drawn from 20,400 respondents from the Survey on Health, Ageing and Retirement in Europe, a panel dataset incorporating a representative sample of the 50+ population in 13 European countries.

Outcomes & analysis: Childhood urbanicity is determined using self-reports of the respondents' circumstances at age 10, and late-adulthood depression using the EURO-D scale. We condition on circumstances early in life as well as later in life, most importantly late-adulthood urbanicity. We estimate the associations using linear regression models and limited dependent variable models.

Results: A pooled regression of both men and women suggests childhood urbanicity is associated non-monotonically with depression in late adulthood, and particularly apparent for those spending their childhoods in suburban settings. We find that individuals that spend the longest time in their childhood in a suburban home exhibit an average increase in probability of 3.4 (*CI* 1.1—5.7) percentage points in reporting four or more depressive symptoms. The association is robust to the inclusion of a host of household characteristics associated with childhood urbanicity, and is independent of current urbanicity and current income. When broken down by gender, we find some evidence of associations between depressive outcomes and urban living for men, and stronger evidence of such associations with urban and suburban living for women, who exhibit an increase of 5.6 (*CI* 2.2—9.0) percentage points in reporting four or more depressive symptoms.

Conclusions: Our analysis reveals a relationship between childhood urbanicity and depression in late-adulthood. The evidence presented on the nature of this relationship is not straightforward but broadly suggestive of a link, differing by gender, between greater urbanicity and higher levels of depressive symptoms. The life-long nature of this association may potentially inform policy agendas aimed at improving urban & suburban living conditions.

Strengths & limitations of this study

- This is the first paper, to the best of our knowledge, to focus on long-term relationships between childhood urbanicity of residence and late-adulthood depressive outcomes specifically.
- We find evidence pointing towards a link between childhood urbanicity and late adulthood depressive outcomes, and highlight potential pathways for this.
- Due to the administrative structure of our data and the absence of any plausible identifying restrictions for causal effects, we are unable to make strong claims regarding causality.

For peer review only

Keywords: urbanisation; depression; mental health; lifecourse / childhood circumstances; ageing.

For peer review only

For peer review only - http://bmjopen.bmj.com/site/about/guidelines.xhtml

INTRODUCTION

The increase in prevalence of mental health disorders in the late 20th and early 21st century, along with increased urban dwelling, raises the question of interplay between these two phenomena. While a substantial body of literature, outlined below, examines links between the lived environment and mental health outcomes at various points over the life course, a potential long-term relationship between childhood urbanicity and late-life depression remains less substantially researched.

A World Health Organisation review[1] reports that an estimated 300 million people suffer from depression globally. Associations with further health conditions and wider adverse socioeconomic outcomes are well-explored, with one meta-analysis[2] evidencing the multiplicity of social costs of major depression, such as reduced educational outcomes, adverse labour market and health outcomes, and increases in other health-damaging behaviours.

As of 2014, 54% of the world's population reside in urban areas, with this figure having risen from 30% since 1950 and being set to rise to 66% by 2050[3]. Various pathways have been suggested for links between levels of urbanicity and health outcomes. Vlahov & Galea [4] report that the urban social environment can affect individuals' general health via certain features such as socioeconomic status, crime and violence, presence of marginalised populations and the prevalence of psychological stressors. Diez Roux & Mair[5] further detail potential pathways for links between physical and social aspects of the lived environment and current health outcomes. Compared to living in a rural area, urban dwellers face an elevated amount of environmental stressors on a daily basis which can lead to long-term degradation of their mental well-being. Further detail of links between city dwelling, quality of life and happiness is provided in [6], with aspects of city dwelling such as pollution, monotony of life, and the artificiality of the city suggested as being potentially detrimental to quality of life. A

For peer review only - http://bmjopen.bmj.com/site/about/guidelines.xhtml

Finnish study[7] suggests that this may be due in part to variation in socioeconomic status between rural and urban areas.

Existing literature has generally focused on the relationships between urbanicity and contemporaneous health outcomes at various stages of the life course, with a number of these covering common mental disorders such as depression[8–13]. While the authors caution against drawing causal conclusions, in a broad meta-analysis of the link between urbanicity and mental health disorders, Penkalla & Kohler[14] find links between the degree of urbanicity and mood and anxiety disorders, psychosis, and substance use disorders. The largest single study[15], using administrative data collected on the entire Swedish population of 4.4 million people aged 25-64, finds that those in the top quintile by urbanicity have a 68-77% higher risk of developing psychosis and 12-20% higher risk of developing depression than those in the lowest, with these relationships being stronger for women than men. Oliver[16] discusses potential links between current suburban and urban living and adverse mental health outcomes.

The relationship between childhood urbanicity and depression later in life remains a topic substantially less explored in existing research. While some evidence [17,18] points to relationships between early life urban dwelling and impacts on brain areas associated with negative affect, we believe there to exist only one study[19] to attempt to carry out preliminary investigations into longer-term and later-life relationships that directly use measures of depression. Literature on the life course perspective[20–23] suggests, however, theoretical roles for the impact of conditions throughout an individual's life (in particular of early life conditions) on subsequent health, with this impact likely to vary in particular by gender (as evidenced by theoretical and empirical reviews[24,25]). Various pathways have been proposed to explain the incidence of depression specifically over the lifecycle, with Rudenstine[26] emphasising a role for the impact of early life circumstances.

For peer review only - http://bmjopen.bmj.com/site/about/guidelines.xhtml

Previous studies examining longer-arm relationships between urbanicity and mental ill-health suggest empirical support for these theoretical pathways. One study employing the records of over 2.3 million people born in Sweden between 1967 and 1989[27], finds that the population density of area of residence at age 15 is predictive of subsequent schizophrenia. Kim, Stewart, Shin, Yoon, & Lee[19] use the principal areas that participants lived in during early-life (up to age 20) and mid-life (21-60) to investigate the relationship between the urbanicity of residence at such ages and depression later in life. The study finds links between current urbanicity and current depressive outcomes, and a greater prevalence of depression among women. Individuals who lived their entire life in rural areas are found to have the lowest rates of depression, with similar rates of depression observed among individuals who grew up in rural areas and subsequently lived in urban areas, and those who spent their entire life in urban areas. Lederbogen et al[17], using magnetic resonance imaging to examine brain activity, present evidence of relationships between urban dwelling in childhood and social stress processing. A further study[28] uses German and Chinese data to investigate links between childhood urbanicity and emotional characteristics later in (mostly young) adulthood. These results, drawn from relatively small samples (324 individuals in Germany, 713 in China) provide some evidence suggestive of a relationship with negative emotional traits for Chinese women alone, among whom *lower* symptoms of "fear" are exhibited for those who lived in urban settings in childhood.

In this paper, we seek to investigate specifically the relationship between early-life urban living and the presence of depression in late adulthood. We employ a cross-country European dataset of individuals aged 50 and above in order to investigate such a relationship, using individuals' self-reports of childhood circumstances, and depressive symptoms as measured by the EURO-D questionnaire. Section 2 introduces our data source and variables used in our study. Section 3 discusses the results of our study and its implications. Section 4 concludes.

For peer review only - http://bmjopen.bmj.com/site/about/guidelines.xhtml

METHODS

Data and sample

For the purpose of our study we employ two waves of the Survey of Health, Ageing and Retirement in Europe (SHARE), a pan-European survey containing information on health, socio-economic and social and family networks of individuals that are aged 50 or older in thirteen European countries plus Israel[1]. SHARE consists of six published waves to date, with individuals tracked across successive surveys. The study design and sampling methods, both in general and for the specific waves here employed, are detailed by the study's authors in several publications[29–33]. A declaration relating to ethical approval is available online [34], and no public or patient involvement beyond this took place. We employ the second contemporaneous wave of the study (collected in 2006-7) and SHARELIFE, the third wave of the survey (collected in 2008-9), which focuses on the self-reported life histories of respondents. In total, 25,341 individuals appear in both datasets. After listwise removal of individuals with missing data for individual variables, our final sample stands at 20,400 (9029 men and 11,371 women) individuals[2], and 13,548 (6,154 men and 7,394 women) when control is made for current urbanicity. A summary of item missingness is shown in Table A1. Table 1 shows the summary statistics for the final sample used in the study, broken down by gender and by childhood urbanicity.

Variables

The main dependent variable used in this study is depression, with symptoms measured by the EURO-D scale included in the SHARE questionnaire. The EURO-D is a symptoms scale for depression and has been validated in a cross-European study[35]. The scale covers 12

---

[1]Israel is, however, not included in our analysis due to its absence from wave 3.
[2]We also use a larger sample (consisting of 24,854 individuals) for our full information maximum likelihood analysis, containing all individuals in both waves of the data with a recorded EURO-D score.

For peer review only - http://bmjopen.bmj.com/site/about/guidelines.xhtml

symptoms: depressed mood, pessimism, suicidality, guilt, sleep, interest, irritability, appetite, fatigue, concentration, enjoyment and tearfulness. The final EURO-D score (between 0 and 12) is calculated from the total number of these symptoms present, with a score of 4 or more found to be an optimal cut-off point for the prediction of depression[35,36]. In addition to a set of models which treat this EURO-D score as the dependent variable in OLS regressions, in a second set of models, we transform this variable into a dichotomous variable that is equal to 1 for EURO-D scores 4 and higher, and 0 for scores strictly below 4.

The main independent variable of interest in our study is the urbanicity of the respondents' childhood homes. In the retrospective SHARELIFE questionnaire, respondents provided information on each dwelling they had lived in since birth, providing the exact year in which they moved, and the type of area in which each dwelling was located. This area type is coded in SHARELIFE with values of 1 ("A big city"), 2 ("The suburbs or outskirts of a big city"), 3 ("A large town"), 4 ("A small town"), 5 ("A rural area or village"). A previous study investigating the recall of other childhood conditions in SHARE [37] presents evidence that individuals recall these in general accurately.

Durations, measured in years, between moving into and out of each dwelling were computed to determine the length that respondents lived in each of these. We then identified the home that respondents lived in for the longest time between birth and the age of 15, and the corresponding area type of that dwelling. In our base case, we include this variable as is, after the exclusion of cases for non-response.

Statistical analysis

We outline here the various statistical methods employed. We estimate our models primarily as a series of ordinary least squares (OLS) and logistic regressions – the former being used for regressions using the EURO-D score as dependent variable and the latter for those using a

For peer review only - http://bmjopen.bmj.com/site/about/guidelines.xhtml

dichotomised EURO-D variable equal to 1 for scores of 4 or more, and to 0 for scores strictly fewer than 4. We also include a full information maximum likelihood (FIML) specification, which allows us to estimate parameters using all available data in the sample under the assumption that observations are missing at random[38,39], as a final robustness check.

We include basic demographic controls of gender and age-period-cohort in all models, as well as allowing for country fixed effects. In summarising the varying social, marital and economic positions of men and women and the potential tendencies of each to respond to adverse life events in different ways, existing literature[24,40] suggests there may be differences both in overall prevalence of depression by gender, and also in relationships between explanatory factors and such mental health outcomes. Furthermore, while all respondents in the SHARELIFE survey are aged above 50, existing literature points to a role for age as a relevant control variable even among this group[41,42]. Although interpretation of the variation explained by age variables in studies such as this is plagued by problems in separately identifying age-period-cohort effects – that is, due to collinearity, identifying these three effects independently is impossible without resorting to additional, untestable, assumptions[43] – we include these variables purely as controls for the combination of all three potential such effects. While for brevity we subsequently refer to these variables as "age" controls, we remain agnostic as to the actual source of variation by age-period-cohort and merely attempt to control for any such relationship. In an initial parsimonious model, we control only for these basic demographic factors.

In further models, we also include additional control variables related to childhood circumstances and characteristics, and contemporaneous log income[3]. Latham[46] reports that childhood disability is significantly associated with later-life depression, a relationship found

[3]We implement this by dividing gross household income by the square root of household size, as per the current method employed by recent OECD publications[44,45].

For peer review only - http://bmjopen.bmj.com/site/about/guidelines.xhtml

to be of an indirect nature through social and health factors such as midlife physical health. To control for such an effect in our model, we introduce self-reported childhood health status, a score between 1 (poor) to 5 (excellent). We also seek to control for childhood socioeconomic circumstances and, to this end, follow a validated method[37] employed in other papers[47,48] employing SHARE, a principal components score, created from a combination of measures of childhood (age 10) socioeconomic status included in SHARELIFE. Table A2 in the Appendix provides the results of our principal component analysis. We use only component 1 to construct the score used in our index of childhood SES, which we divide into deciles in our model. We also add parental presence (split by step- and biological parent) in the childhood home at the age of ten, self-perceived and self-recalled ability at age ten in mathematics and literacy compared to the average and self-reported parental alcohol abuse.

In a final model, we seek to consider the relationship between depression in late adulthood and childhood urbanicity once control is made for current urbanicity at the time at which the SHARE interview was conducted. To this end, we include a variable for current urbanicity coded in an identical way to that in which we code childhood urbanicity, as earlier described.

We also present alternative specifications, changing the dependent variable to the urbanicity of area of residence when aged between 0-5, 6-10, and 11-15 years.

For peer review only

For peer review only - http://bmjopen.bmj.com/site/about/guidelines.xhtml

RESULTS

[Table 1 here]

Out of the 20,400 individuals included in our main sample, 23% recorded a EURO-D score of 4 or above, with an average score of 2.18. We find that women (29%) were almost twice as likely as men (15%) to exhibit a EURO-D score of 4 or above, and this score for women (2.58) was just under one point higher on average than for men (1.68).

[Tables 2-4 here]

Our results suggest that the urbanicity of childhood residence, rather than of current residence, is of primary importance in the relationship between the lived environment and depression. These results also suggest that such a relationship varies by gender, with an F-test of the interactions between gender and childhood urbanicity categories being rejected at $p < 0.0001$. A matrix of individuals' urbanicity of residence in childhood and currently is presented in Table A3.

Our results suggest a complex relationship between level of childhood urbanicity and later life depression. A parsimonious pooled regression (Column 1, Table 2) including both men and women suggests that, compared to individuals living in rural areas or villages, only individuals spending their childhoods in the suburbs of big cities are observed to have significantly worse outcomes in late adulthood. Our model with childhood controls (Column 2, Table 2) suggests that individuals growing up in big cities are observed to have EURO-D scores of 0.0978 ($p < 0.05$) on average worse than those growing up in rural areas or villages. In the same model, individuals growing up in the suburbs of big cities have worse still depressive outcomes as

For peer review only - http://bmjopen.bmj.com/site/about/guidelines.xhtml

measured by the EURO-D score, with this score being on average 0.153 ($p < 0.01$) higher for such individuals. Individuals growing up in large towns are estimated to have a EURO-D score in adulthood on average 0.0760 ($p < 0.10$) higher than those growing up in rural areas. These results are robust to respecification of the model with our age variable modified to, variously, the type of area lived for longest between the ages of 0-5, 6-10, 11-15 (Table A4).

When broken down by gender, these relationships across our models appear to differ substantially. Much stronger results are observed for women than for men. While only the coefficient for having spent childhood in a big city reaches conventional levels of significance for men (Column 4, Table 2), this relationship is only observed for women for those who grew up in the suburbs of a big city (Column 6, Table 2). Men with childhoods spent in a big city are estimated to have a EURO-D score of on average 0.104 ($p < 0.10$) higher than those who grew up in rural areas or villages, while women with suburban childhoods are observed to have a EURO-D score of on average 0.223 ($p < 0.01$) higher than those who grew up in rural areas or villages.

While these may appear to represent small increases, it is instructive to also consider the results of a logit model with presence of a EURO-D score of 4 or more as dependent variable, with these results presented in Table 3 (odds-ratios) and Table 4 (average marginal effects). Such a model again suggests that suburban childhoods are associated with worse depressive outcomes for women, with an average marginal effect estimated for women of 5.58 percentage points in our preferred model.

While we confirm a role for childhood urbanicity in explaining adult urbanicity (with an F-test of the relevance of such variables in an ordered logit model with adulthood urbanicity as the dependent variable being rejected at $p < 0.0001$), the addition of this as an explanatory variable

For peer review only - http://bmjopen.bmj.com/site/about/guidelines.xhtml

(Columns 3, 5 & 7, Table 2) does not substantially affect our estimated relationship between urbanicity of childhood residence and the individual's EURO-D score in late adulthood[4].

For peer review only

[4] Although there is a reduction in statistical significance (indeed a loss of significance for our big city point estimate), it is worth noting that the addition of current urbanicity reduces our sample size by approximately one third. None of our point estimates are substantially affected by the addition of current urbanicity.

For peer review only - http://bmjopen.bmj.com/site/about/guidelines.xhtml

DISCUSSION

These results are suggestive of a long–term relationship, persisting beyond the age of 50, between levels of childhood urbanicity and depression. The presence of such a relationship is robust to several model specifications, and is most commonly observed for those who grew up in suburban areas. Contra existing literature, we find that increasing levels of contemporaneous urbanicity generally have a small and non-significant negative relationship with both EURO-D score (in our linear regressions) and presence of a EURO-D score of 4 or more (in our logistic regressions). Furthermore, the inclusion of this additional variable does not substantially impact upon the magnitude of our parameter estimates of the relationship between childhood urbanicity and depressive outcomes. These small and statistically insignificant parameter estimates suggest little evidence of a relationship between current urbanicity and the presence of depression in our sample.

With the continued trend of rapid urbanisation and the rising prominence of mental health conditions[1,3], the investigation of links between childhood urbanicity and mental health outcomes is required to help policy makers better understand and design policies to cater to such growing needs. Our study follows in the footsteps of existing literature on urbanicity and mental health but goes on to specifically look at the relationship of urbanicity and depression from a long-term standpoint, as well as being, to our knowledge, the first of this type to consider separate relationships by gender. In contrast to existing studies in the field, our study employs a cross-national sample with a comprehensive and wide range of retrospective data.

In line with literature in the life course perspective emphasising the importance of early life circumstances, we find that spending one's childhood in an urban or suburban area has a positive and significant relationship with the presence of depression in later-life adulthood when a pooled regression is run for the entire sample. This relationship persists even after

For peer review only - http://bmjopen.bmj.com/site/about/guidelines.xhtml

controlling for other socioeconomic, demographic and environmental factors. This relationship appears to be particularly strong for women. Individuals that spend the longest time in their childhood in a suburban home face an average increase in probability of 3.4 percentage points (5.6 points for women) of reporting four or more depressive symptoms on the EURO-D scale during later-life adulthood, a relationship that persists even when control is made for current urbanicity. It is important to acknowledge that the nature of SHARE is such that going beyond establishing correlations in our data is not possible. While we are unable to establish any identifying restrictions for such causal effects in our dataset, the findings here are striking and establish potential pathways for future research. Although SHARE's rich retrospective information, even if based on recall data, provides a rare opportunity to investigate long-term relationships such as this, the use of linked administrative long panel datasets would be preferable. Such datasets would contain more comprehensive information about specific areas of residence and would help to overcome one limitation of our study: that of treating "big city" as one homogeneous item, borne out of our inability to account for the fact that the experience of city life varies both within and between cities. Future research could seek to overcome some of these inherent limitations of our approach, in seeking to establish causal effects, as well as to establish more clearly theoretical pathways for such effects.

For peer review only

For peer review only - http://bmjopen.bmj.com/site/about/guidelines.xhtml

APPENDIX

[Tables A1-A4 here]

For peer review only - http://bmjopen.bmj.com/site/about/guidelines.xhtml

References

1 World Health Organisation. Mental disorders. WHO. 2017.http://www.who.int/mediacentre/factsheets/fs396/en/ (accessed 7 Aug 2017).

2 Kessler RC. The Costs of Depression. *Psychiatr Clin* 2012;**35**:1–14. doi:10.1016/j.psc.2011.11.005

3 United Nations, Department of Economic and Social Affairs, Population Division. *World Urbanization Prospects, the 2014 Revision: Highlights*. 2014.

4 Vlahov D, Galea S. Urbanization, urbanicity, and health. *J Urban Health Bull N Y Acad Med* 2002;**79**:S1–12. doi:10.1093/jurban/79.suppl_1.S1

5 Diez Roux AV, Mair C. Neighborhoods and health. *Ann N Y Acad Sci* 2010;**1186**:125–45. doi:10.1111/j.1749-6632.2009.05333.x

6 Okulicz-Kozaryn A. *Happiness and Place: Why Life is Better Outside of the City*. 1st ed. 2015 edition. Palgrave Pivot 2015.

7 Airaksinen J, Hakulinen C, Elovainio M, *et al.* Neighborhood effects in depressive symptoms, social support, and mistrust: Longitudinal analysis with repeated measurements. *Soc Sci Med* 2015;**136–137**:10–6. doi:10.1016/j.socscimed.2015.04.034

8 Kim M-H, Jung-Choi K, Jun H-J, *et al.* Socioeconomic inequalities in suicidal ideation, parasuicides, and completed suicides in South Korea. *Soc Sci Med* 2010;**70**:1254–61. doi:10.1016/j.socscimed.2010.01.004

9 Maas J, Verheij RA, Groenewegen PP, *et al.* Green space, urbanity, and health: how strong is the relation? *J Epidemiol Community Health* 2006;**60**:587–92. doi:10.1136/jech.2005.043125

10 Newbury J, Arseneault L, Caspi A, *et al.* Why Are Children in Urban Neighborhoods at Increased Risk for Psychotic Symptoms? Findings From a UK Longitudinal Cohort Study. *Schizophr Bull* 2016;**42**:1372–83. doi:10.1093/schbul/sbw052

11 van de Poel E, O'Donnell O, van Doorslaer E. Urbanization and the spread of diseases of affluence in China. *Econ Hum Biol* 2009;**7**:200–16. doi:10.1016/j.ehb.2009.05.004

12 Weich S, Twigg L, Holt G, *et al.* Contextual risk factors for the common mental disorders in Britain: a multilevel investigation of the effects of place. *J Epidemiol Community Health* 2003;**57**:616–21. doi:10.1136/jech.57.8.616

13 Kovess-Masféty V, Alonso J, de Graaf R, *et al.* A European approach to rural-urban differences in mental health: the ESEMeD 2000 comparative study. *Can J Psychiatry Rev Can Psychiatr* 2005;**50**:926–36. doi:10.1177/070674370505001407

14 Penkalla AM, Kohler S. Urbanicity and Mental Health in Europe: A Systematic Review. *Eur J Ment Health* 2014;**9**:163–77.

For peer review only

For peer review only - http://bmjopen.bmj.com/site/about/guidelines.xhtml

15 Sundquist K, Frank G, Sundquist J. Urbanisation and incidence of psychosis and depression: follow-up study of 4.4 million women and men in Sweden. *Br J Psychiatry J Ment Sci* 2004;**184**:293–8.

16 Oliver JE. Mental Life and the Metropolis in Suburban America: The Psychological Correlates of Metropolitan Place Characteristics. *Urban Aff Rev* 2003;**39**:228–53. doi:10.1177/1078087403254445

17 Lederbogen F, Kirsch P, Haddad L, *et al.* City living and urban upbringing affect neural social stress processing in humans. *Nature* 2011;**474**:498–501. doi:10.1038/nature10190

18 Pezawas L, Meyer-Lindenberg A, Drabant EM, *et al.* 5-HTTLPR polymorphism impacts human cingulate-amygdala interactions: a genetic susceptibility mechanism for depression. *Nat Neurosci* 2005;**8**:828. doi:10.1038/nn1463

19 Kim J-M, Stewart R, Shin I-S, *et al.* Lifetime urban/rural residence, social support and late-life depression in Korea. *Int J Geriatr Psychiatry* 2004;**19**:843–51. doi:10.1002/gps.1175

20 Ben-Shlomo Y, Kuh D. A life course approach to chronic disease epidemiology: conceptual models, empirical challenges and interdisciplinary perspectives. *Int J Epidemiol* 2002;**31**:285–93. doi:10.1093/ije/31.2.285

21 Koenen KC, Rudenstine S, Susser E, *et al.*, editors. *A Life Course Approach to Mental Disorders*. Oxford, New York: : Oxford University Press 2013.

22 Kuh D, Ben Shlomo Y, editors. *A Life Course Approach to Chronic Disease Epidemiology*. Second Edition. Oxford, New York: : Oxford University Press 2004.

23 Pudrovska T, Anikputa B. Early-life socioeconomic status and mortality in later life: an integration of four life-course mechanisms. *J Gerontol B Psychol Sci Soc Sci* 2014;**69**:451–60. doi:10.1093/geronb/gbt122

24 Cleary PD. Gender differences in stress-related disorders. In: Barnett RC, Biener L, Baruch GK, eds. *Gender and stress*. New York, NY, US: : Free Press 1987. 39–72.

25 Van de Velde S, Bracke P, Levecque K. Gender differences in depression in 23 European countries. Cross-national variation in the gender gap in depression. *Soc Sci Med 1982* 2010;**71**:305–13. doi:10.1016/j.socscimed.2010.03.035

26 Rudenstine S. Applying a life course perspective to depression. In: Koenen KC, Rudenstine S, Susser E, *et al.*, eds. *A Life Course Approach to Mental Disorders*. Oxford University Press 2013. 88–96. doi:10.1093/acprof:oso/9780199657018.003.0008

27 Sariaslan A, Larsson H, D'Onofrio B, *et al.* Does Population Density and Neighborhood Deprivation Predict Schizophrenia? A Nationwide Swedish Family-Based Study of 2.4 Million Individuals. *Schizophr Bull* 2015;**41**:494–502. doi:10.1093/schbul/sbu105

28 Sindermann C, Kendrick KM, Becker B, *et al.* Does Growing up in Urban Compared to Rural Areas Shape Primary Emotional Traits? *Behav Sci Basel Switz* 2017;**7**. doi:10.3390/bs7030060

For peer review only

29 Börsch-Supan A. Survey of Health, Ageing and Retirement in Europe (SHARE) Wave 2. 2017. doi:10.6103/SHARE.w2.600

30 Börsch-Supan A. Survey of Health, Ageing and Retirement in Europe (SHARE) Wave 3 - SHARELIFE. 2017. doi:10.6103/SHARE.w3.600

31 Börsch-Supan A, Brugiavini A, Jürges H, *et al. First Results from the Survey of Health, Ageing, and Retirement in Europe (2004-2007) : Starting the longitudinal dimension*. Mannheim: : Mannheim Research Institute for the Economics of Aging 2008. http://www.share-project.org/fileadmin/pdf_documentation/FRB2/FRB2_all_chapters.pdf (accessed 11 Apr 2017).

32 Börsch-Supan A, Brandt M, Hunkler C, *et al.* Data Resource Profile: the Survey of Health, Ageing and Retirement in Europe (SHARE). *Int J Epidemiol* 2013;**42**:992–1001. doi:10.1093/ije/dyt088

33 Börsch-Supan A, Schröder M. Retrospective data collection in the Survey of Health, Ageing and Retirement in Europe. *SHARELIFE Methodol* 2011;**5**.http://www.share-project.org/uploads/tx_sharepublications/FRB-Methodology_feb2011_color-1.pdf#page=6

34 Wolfrum R. Opinion of the Ethics Council of the Max Planck Society on the 'SHARE' Project. 2016.http://www.share-project.org/fileadmin/pdf_documentation/SHARE_ethics_approvals.pdf

35 Prince MJ, Reischies F, Beekman AT, *et al.* Development of the EURO-D scale--a European, Union initiative to compare symptoms of depression in 14 European centres. *Br J Psychiatry* 1999;**174**:330–8. doi:10.1192/bjp.174.4.330

36 Prince MJ, Beekman AT, Deeg DJ, *et al.* Depression symptoms in late life assessed using the EURO-D scale. Effect of age, gender and marital status in 14 European centres. *Br J Psychiatry J Ment Sci* 1999;**174**:339–45.

37 Havari E, Mazzonna F. Can We Trust Older People's Statements on Their Childhood Circumstances? Evidence from SHARELIFE. *Eur J Popul* 2015;**31**:233–57. doi:10.1007/s10680-014-9332-y

38 Schafer JL, Graham JW. Missing data: Our view of the state of the art. *Psychol Methods* 2002;**7**:147–77. doi:10.1037/1082-989X.7.2.147

39 Enders CK. The Performance of the Full Information Maximum Likelihood Estimator in Multiple Regression Models with Missing Data. *Educ Psychol Meas* 2001;**61**:713–40. doi:10.1177/0013164401615001

40 Simon RW. Revisiting the relationships among gender, marital status, and mental health. *AJS* 2002;**107**:1065–96.

41 Keyes M, Legrand LN, Iacono WG, *et al.* Parental Smoking and Adolescent Problem Behavior: An Adoption Study of General and Specific Effects. *Am J Psychiatry* 2008;**165**:1338–44. doi:10.1176/appi.ajp.2008.08010125

For peer review only - http://bmjopen.bmj.com/site/about/guidelines.xhtml

42 Mirowsky J, Ross CE. Age and Depression. *J Health Soc Behav* 1992;**33**:187–205. doi:10.2307/2137349

43 Bell A, Jones K. The impossibility of separating age, period and cohort effects. *Soc Sci Med 1982* 2013;**93**:163–5. doi:10.1016/j.socscimed.2013.04.029

44 Organisation for Economic Co-operation and Development. Divided We Stand: Why Inequality Keeps Rising: An overview of growing income inequalities in OECD countries: main findings. *OECD Publ* Published Online First: 2011.http://www.ingentaconnect.com/content/oecd/16815378/2011/00002011/00000014/8111111ec003

45 Organisation for Economic Co-operation and Development. What are equivalence scales? *OECD Proj Income Distrib Poverty* 2013.

46 Latham K. The 'long arm' of childhood health: linking childhood disability to late midlife mental health. *Res Aging* 2015;**37**:82–102. doi:10.1177/0164027514522276

47 Angelini V, Mierau J. Late-life health effects of teenage motherhood. *Demogr Res* 2018;**39**:1081–104. doi:10.4054/DemRes.2018.39.41

48 Angelini V, Howdon DDD, Mierau JO. Childhood Socioeconomic Status and Late-Adulthood Mental Health: Results from the Survey on Health, Ageing and Retirement in Europe. *J Gerontol B Psychol Sci Soc Sci* Published Online First: 16 March 2018. doi:10.1093/geronb/gby028

For peer review only

Tables

TABLE 1
Descriptive Statistics (N = 20,400)

Name	All	All	Mean / % Men	Mean / % Women	
Individual Characteristics			(N=9,029)	(N=11,371)	
Male (N, %)	9,029	*44.26*	N/A	N/A	
Age (mean, *sd*)	64.23	*9.43*	64.34	64.15	
Urbanicity of current residence (mean, *sd*)	3.33	*1.41*	3.35	3.31	
Urbanicity of childhood residence (mean, *sd*)	3.70	1.46	3.67	3.71	
Equivalised HH income (000s) (mean, *sd*)	21.57	*30.69*	23.36	20.16	***
Health Status					
Depression (EURO-D)	2.18	*2.15*	1.68	2.58	***
EURO-D score 4+ (N, %)	4,666	*22.87*	*14.67*	*29.38*	***
Childhood SES					
Number of Rooms (mean, *sd*)	3.72	*1.79*	3.73	3.71	
Number of Facilities (mean, *sd*)	2.28	*1.48*	2.28	2.28	
Number of Books (1 = few to 5 = >200 books) (mean, *sd*)	2.13	*1.21*	2.11	2.14	
Occupation of Main Breadwinner (ranked) (mean, *sd*)	2.90	*0.80*	2.90	2.91	
Childhood Characteristics					
Math Performance (1 = low to 5 = high) (mean, *sd*)	3.30	*0.89*	3.39	3.23	***
Language Performance (1 = low to 5 = high) (mean, *sd*)	3.33	*0.87*	3.24	3.41	***
Self-Reported Health (1 = poor to 5 = excellent) (mean, *sd*)	3.97	*1.00*	4.04	3.91	***
Parent heavy drinker (N, %)	1,658	*8.13*	*8.09*	*8.16*	
Biological Father Present (N, %)	18,582	*91.09*	*91.15*	*91.04*	
Biological Mother Present (N, %)	19,639	*96.27*	*3.41*	*3.98*	*
Stepfather Present (N, %)	441	*2.16*	*2.10*	*2.21*	
Stepmother Present (N, %)	322	*1.58*	*1.46*	*1.67*	

Note: */**/*** indicate significant differences at the 10%/5%/1% level based on Student's t-tests of group mean comparisons.

For peer review only - http://bmjopen.bmj.com/site/about/guidelines.xhtml

TABLE 2

Association Between Childhood Urbanicity and Late-Adulthood EURO-D score (OLS coefficients)

		Full Sample		Men	Men	Women	Women
Dependent variable	EURO-D	EURO-D	EURO-D	EURO-D	EURO-D	EURO-D	EURO-D
	(1)	(2)	(3)	(4)	(5)	(6)	(7)
Age 0-15 living environment (omitted: rural area or village)							
Big city	0.0474	0.0978**	0.117**	0.104*	0.0755	0.0937	0.151*
	(0.0432)	(0.0440)	(0.0566)	(0.0582)	(0.0740)	(0.0641)	(0.0836)
Suburbs or outskirts of a big city	0.117**	0.153***	0.156**	0.0563	0.0355	0.223***	0.253**
	(0.0560)	(0.0562)	(0.0704)	(0.0751)	(0.0913)	(0.0812)	(0.105)
Large town	0.0208	0.0760*	0.0977*	0.0653	0.0812	0.0834	0.124
	(0.0433)	(0.0436)	(0.0558)	(0.0583)	(0.0726)	(0.0631)	(0.0826)
Small town	-0.0275	0.0136	0.0153	0.0818	0.114*	-0.0471	-0.0791
	(0.0412)	(0.0410)	(0.0518)	(0.0552)	(0.0683)	(0.0590)	(0.0759)
Male	-0.887***	-0.846***	-0.864***				
	(0.0286)	(0.0287)	(0.0355)				
Current living environment (omitted: rural area or village)							
Big city			-0.0850		-0.0755		-0.0634
			(0.0603)		(0.0800)		(0.0883)
Suburbs or outskirts of a big city			-0.0528		0.0267		-0.115
			(0.0588)		(0.0759)		(0.0879)
Large town			0.0299		-0.0670		0.136*
			(0.0541)		(0.0715)		(0.0792)
Small town			-0.0840		-0.0868		-0.0669
			(0.0514)		(0.0671)		(0.0760)

For peer review only

Additional childhood controls & contemporaneous income	NO	YES	YES	YES	YES	YES	YES
Observations	20,400	20,400	13,548	9,029	6,154	11,371	7,394

*Notes: Constant not reported. */**/*** indicate statistical significance at the 10%/5%/1% level, standard errors between brackets.*

All models contain controls for age-period-cohort and country fixed effects. Childhood controls consist of self-reported and self-recalled health status, a principal components score for deprivation, self-perceived and self-recalled numeracy and literacy at age 10, and self-reported parental alcohol abuse

Column 1 displays the estimation results from regressing the EURO-D depression score on a set of living conditions when the respondent was aged 0 – 15. Column 2 adds various childhood & contemporaneous controls and Column 3 controls for current living conditions. Columns 4 & 5 and 6 & 7 repeat the specifications of columns 2 & 3 for men and women separately, respectively.

For peer review only

TABLE 3

Association Between Childhood Urbanicity and Late-Adulthood Depression incidence (Logit, odds ratios)

	Full Sample			Men	Men	Women	Women
	(1)	(2)	(3)	(4)	(5)	(6)	(7)
Age 0-15 living environment (omitted: rural area or village)							
Big city	1.045	1.121**	1.100	1.129	1.021	1.117	1.147
	(0.0566)	(0.0634)	(0.0797)	(0.109)	(0.127)	(0.0783)	(0.103)
Suburbs or outskirts of a big city	1.176**	1.235***	1.217**	1.039	0.990	1.334***	1.350***
	(0.0810)	(0.0876)	(0.108)	(0.132)	(0.152)	(0.115)	(0.148)
Large town	1.036	1.109*	1.087	1.084	1.047	1.120*	1.116
	(0.0556)	(0.0615)	(0.0765)	(0.105)	(0.127)	(0.0761)	(0.0976)
Small town	0.996	1.045	1.057	1.106	1.143	1.014	1.007
	(0.0504)	(0.0540)	(0.0685)	(0.0993)	(0.125)	(0.0643)	(0.0812)
Male	0.399***	0.410***	0.416***				
	(0.0149)	(0.0157)	(0.0193)				
Current living environment (omitted: rural area or village)							
Big city			0.947		0.927		0.980
			(0.0716)		(0.121)		(0.0916)
Suburbs or outskirts of a big city			0.984		1.071		0.945
			(0.0737)		(0.132)		(0.0890)
Large town			1.052		0.915		1.155*
			(0.0705)		(0.106)		(0.0961)
Small town			0.871**		0.825*		0.906
			(0.0565)		(0.0908)		(0.0732)
Additional childhood controls & contemporaneous income	NO	YES	YES	YES	YES	YES	YES
Observations	20,400	20,400	13,548	9,029	6,154	11,371	7,394

For peer review only

*Notes: Constant not reported. */**/*** indicate statistical significance at the 10%/5%/1% level, standard errors between brackets.*

All models contain controls for age-period-cohort and country fixed effects. Childhood controls consist of self-reported and self-recalled health status, a principal components score for deprivation, self-perceived and self-recalled numeracy and literacy at age 10, and self-reported parental alcohol abuse

Column 1 displays the estimation results from regressing the EURO-D depression score on a set of living conditions when the respondent was aged 0 – 15. Column 2 adds various childhood & contemporaneous controls and Column 3 controls for current living conditions. Columns 4 & 5 and 6 & 7 repeat the specifications of columns 2 & 3 for men and women separately, respectively.

For peer review only

TABLE 4

Association Between Childhood Urbanicity and Late-Adulthood Depression incidence (Logit, average marginal effects)

	Full Sample			Men	Men	Women	Women
	(1)	(2)	(3)	(4)	(5)	(6)	(7)
Age 0-15 living environment (omitted: rural area or village)							
Big city	0.00717	0.0181**	0.0151	0.0144	0.00240	0.0208	0.0261
	(0.00878)	(0.00903)	(0.0116)	(0.0117)	(0.0147)	(0.0133)	(0.0173)
Suburbs or outskirts of a big city	0.0268**	0.0340***	0.0319**	0.00443	-0.00118	0.0558***	0.0586***
	(0.0117)	(0.0118)	(0.0147)	(0.0148)	(0.0179)	(0.0172)	(0.0220)
Large town	0.00569	0.0162*	0.0132	0.00944	0.00546	0.0212*	0.0209
	(0.00868)	(0.00881)	(0.0112)	(0.0115)	(0.0144)	(0.0129)	(0.0167)
Small town	-0.000625	0.00683	0.00875	0.0118	0.0163	0.00253	0.00129
	(0.00806)	(0.00806)	(0.0102)	(0.0107)	(0.0136)	(0.0117)	(0.0150)
Male	-0.148***	-0.141***	-0.140***				
	(0.00580)	(0.00583)	(0.00714)				
Current living environment (omitted: rural area or village)							
Big city			-0.00870		-0.00923		-0.00388
			(0.0120)		(0.0157)		(0.0178)
Suburbs or outskirts of a big city			-0.00255		0.00866		-0.0108
			(0.0120)		(0.0157)		(0.0178)
Large town			0.00823		-0.0107		0.0282*
			(0.0109)		(0.0139)		(0.0163)
Small town			-0.0216**		-0.0225*		-0.0185
			(0.0101)		(0.0128)		(0.0151)

For peer review only - http://bmjopen.bmj.com/site/about/guidelines.xhtml

For peer review only

Additional childhood controls & contemporaneous income	NO	YES	YES	YES	YES	YES	YES
Observations	20,400	20,400	13,548	9,029	6,154	11,371	7,394

*Notes: Constant not reported. */**/*** indicate statistical significance at the 10%/5%/1% level, standard errors between brackets.*

All models contain controls for age-period-cohort and country fixed effects. Childhood controls consist of self-reported and self-recalled health status, a principal components score for deprivation, self-perceived and self-recalled numeracy and literacy at age 10, and self-reported parental alcohol abuse.

Column 1 displays the estimation results from regressing the EURO-D depression score on a set of living conditions when the respondent was aged 0 – 15. Column 2 adds various childhood & contemporaneous controls and Column 3 controls for current living conditions. Columns 4 & 5 and 6 & 7 repeat the specifications of columns 2 & 3 for men and women separately, respectively.

For peer review only

TABLE A1
Item missingness: summary (all individuals in both waves, n=25,341)

Variable	Missing count	Present count
EURO-D	487	24,854
Childhood urban residence	1,715	23,626
Age	716	24,625
Childhood maths performance	859	24,482
Childhood language performance	927	24,414
Childhood self-reported health	189	25,152
Parent heavy drinker	103	25,238
Deprivation score	1,172	24,169
Biological Father Present	506	24,835
Biological Mother Present	506	24,835
Stepfather Present	506	24,835
Stepmother Present	506	24,835
Equivalised HH income	444	24,897
Current urban residence	8,882	16,459

For peer review only

TABLE A2

Principal component analysis for childhood SES

	Component			
	1st	2nd	3rd	4th
Number of Rooms	0.4073	0.8637	-0.2968	-0.0111
Number of Books (grouping)	0.5651	-0.1752	0.2367	0.7707
Occupation of Main Breadwinner (ranked)	-0.4758	0.4695	0.7042	0.2394
Number of Facilities	0.537	-0.0546	0.6	-0.5905

For peer review only

TABLE A3

Urbanicity of childhood residence and of current residence (all values percentages)

		Current residence					
		1	2	3	4	5	Total
Childhood residence	1	37.65	21.28	15.13	11.31	14.63	100
	2	17.19	39.64	14.03	14.84	14.30	100
	3	14.39	16.20	38.87	16.81	13.73	100
	4	11.14	12.62	20.40	38.10	17.75	100
	5	9.31	9.05	15.80	21.98	43.85	100
	Total	15.23	15.04	19.74	21.89	28.11	100

Key

1: A big city

2: The suburbs or outskirts of a big city

3: A large town

4: A small town

5: A rural area or village

For peer review only

TABLE A4

Association Between Stage of Childhood Urbanicity and Late-Adulthood Depression Outcomes

	OLS			Logit, average marginal effects		
	(1)	(2)	(3)	(4)	(5)	(6)
Age	0-5	6-10	11-15	0-5	6-10	11-15
Childhood living environment (omitted: rural area or village)						
Big city	0.0913**	0.104**	0.124***	0.0193**	0.0215**	0.0234***
	(0.0437)	(0.0440)	(0.0439)	(0.00889)	(0.00897)	(0.00893)
Suburbs or outskirts of a big city	0.173***	0.159***	0.194***	0.0234**	0.0209*	0.0152
	(0.0577)	(0.0562)	(0.0554)	(0.0117)	(0.0114)	(0.0114)
Large town	0.0706	0.0781*	0.0977**	0.00527	0.00831	0.00729
	(0.0439)	(0.0437)	(0.0434)	(0.00915)	(0.00912)	(0.00905)
Small town	-0.0119	0.0165	0.0187	-0.00171	0.00242	-0.00300
	(0.0410)	(0.0410)	(0.0409)	(0.00874)	(0.00874)	(0.00875)
Male	-0.845***	-0.845***	-0.845***	-0.121***	-0.121***	-0.121***
	(0.0287)	(0.0287)	(0.0287)	(0.00583)	(0.00583)	(0.00583)
Observations	20,400	20,400	20,400	20,400	20,400	20,400

*Notes: Constant not reported. */**/*** indicate statistical significance at the 10%/5%/1% level, standard errors between brackets.*

All models contain controls for age-period-cohort, country fixed effects and additional childhood controls & contemporaneous income.

For peer review only

STROBE 2007 (v4) checklist of items to be included in reports of observational studies in epidemiology*
Checklist for cohort, case-control, and cross-sectional studies (combined)

Section/Topic	Item #	Recommendation	Reported on page #
**Title and abstract**	1	(*a*) Indicate the study's design with a commonly used term in the title or the abstract	3
		(*b*) Provide in the abstract an informative and balanced summary of what was done and what was found	3
**Introduction**			
Background/rationale	2	Explain the scientific background and rationale for the investigation being reported	4-6
Objectives	3	State specific objectives, including any pre-specified hypotheses	6
**Methods**			
Study design	4	Present key elements of study design early in the paper	7-10
Setting	5	Describe the setting, locations, and relevant dates, including periods of recruitment, exposure, follow-up, and data collection	7
Participants	6	(*a*) *Cohort study*—Give the eligibility criteria, and the sources and methods of selection of participants. Describe methods of follow-up<br>*Case-control study*—Give the eligibility criteria, and the sources and methods of case ascertainment and control selection. Give the rationale for the choice of cases and controls<br>*Cross-sectional study*—Give the eligibility criteria, and the sources and methods of selection of participants	7
		(*b*) *Cohort study*—For matched studies, give matching criteria and number of exposed and unexposed<br>*Case-control study*—For matched studies, give matching criteria and the number of controls per case	n/a
Variables	7	Clearly define all outcomes, exposures, predictors, potential confounders, and effect modifiers. Give diagnostic criteria, if applicable	7-10
Data sources/ measurement	8*	For each variable of interest, give sources of data and details of methods of assessment (measurement). Describe comparability of assessment methods if there is more than one group	*7-10*
Bias	9	Describe any efforts to address potential sources of bias	8-9
Study size	10	Explain how the study size was arrived at	7 (also Table A3)
Quantitative variables	11	Explain how quantitative variables were handled in the analyses. If applicable, describe which groupings were chosen and why	7-10
Statistical methods	12	(*a*) Describe all statistical methods, including those used to control for confounding	7-10
		(*b*) Describe any methods used to examine subgroups and interactions	11
		(*c*) Explain how missing data were addressed	7, 9
		(*d*) *Cohort study*—If applicable, explain how loss to follow-up was addressed<br>*Case-control study*—If applicable, explain how matching of cases and controls was addressed	7, 9

For peer review only

		Cross-sectional study—If applicable, describe analytical methods taking account of sampling strategy	
		(*e*) Describe any sensitivity analyses	9-10
**Results**			
Participants	13*	(a) Report numbers of individuals at each stage of study—eg numbers potentially eligible, examined for eligibility, confirmed eligible, included in the study, completing follow-up, and analysed	7
		(b) Give reasons for non-participation at each stage	Table A3
		(c) Consider use of a flow diagram	n/a
Descriptive data	14*	(a) Give characteristics of study participants (eg demographic, clinical, social) and information on exposures and potential confounders	7
		(b) Indicate number of participants with missing data for each variable of interest	Table A3
		(c) *Cohort study*—Summarise follow-up time (eg, average and total amount)	
Outcome data	15*	*Cohort study*—Report numbers of outcome events or summary measures over time	
		Case-control study—Report numbers in each exposure category, or summary measures of exposure	
		Cross-sectional study—Report numbers of outcome events or summary measures	7, 21
Main results	16	(*a*) Give unadjusted estimates and, if applicable, confounder-adjusted estimates and their precision (eg, 95% confidence interval). Make clear which confounders were adjusted for and why they were included	22-27
		(*b*) Report category boundaries when continuous variables were categorized	7-8
		(*c*) If relevant, consider translating estimates of relative risk into absolute risk for a meaningful time period	n/a
Other analyses	17	Report other analyses done—eg analyses of subgroups and interactions, and sensitivity analyses	24-27
**Discussion**			
Key results	18	Summarise key results with reference to study objectives	14
Limitations	19	Discuss limitations of the study, taking into account sources of potential bias or imprecision. Discuss both direction and magnitude of any potential bias	15
Interpretation	20	Give a cautious overall interpretation of results considering objectives, limitations, multiplicity of analyses, results from similar studies, and other relevant evidence	15
Generalisability	21	Discuss the generalisability (external validity) of the study results	15
**Other information**			
Funding	22	Give the source of funding and the role of the funders for the present study and, if applicable, for the original study on which the present article is based	n/a

*Give information separately for cases and controls in case-control studies and, if applicable, for exposed and unexposed groups in cohort and cross-sectional studies.
Note: An Explanation and Elaboration article discusses each checklist item and gives methodological background and published examples of transparent reporting. The STROBE checklist is best used in conjunction with this article (freely available on the Web sites of PLoS Medicine at http://www.plosmedicine.org/, Annals of Internal Medicine at http://www.annals.org/, and Epidemiology at http://www.epidem.com/). Information on the STROBE Initiative is available at www.strobe-statement.org.

For peer review only

BMJ Open

The Relationship Between Early Life Urbanicity and Depression in Late-Adulthood: Evidence from the Survey of Health, Ageing and Retirement in Europe

Journal:	*BMJ Open*
Manuscript ID	bmjopen-2018-028090.R2
Article Type:	Research
Date Submitted by the Author:	31-Jul-2019
Complete List of Authors:	Howdon, Daniel; University of Leeds Leeds Institute of Health Sciences, Academic Unit of Health Economics<br>Mierau, Jochen; Rijksuniversiteit Groningen Faculteit Economie en Bedrijfskunde, Economics, Econometrics and Finance<br>Liew, Samuel; Copenhagen Business School
**Primary Subject Heading**:	Public health
Secondary Subject Heading:	Health economics, Mental health
Keywords:	MENTAL HEALTH, HEALTH ECONOMICS, PUBLIC HEALTH

SCHOLARONE™
Manuscripts

The Relationship Between Early Life Urbanicity and Depression in Late-Adulthood: Evidence from the Survey of Health, Ageing and Retirement in Europe

Daniel Howdon [1, 2] Jochen O. Mierau [*, 2, 3, 4] Samuel K. M. Liew

* Corresponding author. Department of Economics, Econometrics and Finance, Faculty of Economics and Business, University of Groningen, Nettelbosje 2, 9747AE Groningen, The Netherlands. E-mail: j.o.mierau@rug.nl.

1: Academic Unit of Health Economics, Leeds Institute of Health Sciences, University of Leeds, UK

2: Department of Economics, Econometrics and Finance, Faculty of Economics and Business, University of Groningen, Groningen, Netherlands

3: NETSPAR, Tilburg, Netherlands

4: Aletta Jacobs School of Public Health, University of Groningen, Groningen, Netherlands.

Word count: 3509

For peer review only

Authorship statement:

SL carried out initial empirical estimation and wrote the first draft as a postgraduate thesis under the supervision of DH and JM. DH carried out re-estimation and estimation of additional model specifications, and substantially redrafted the paper. JM and SL made further redrafting contributions to the paper. All authors approved the final draft.

Data sharing:

The data is a panel dataset available from the SHARE website, with licensing subject to use in scientific research.

Quoting directly from the dataset's website:

http://www.share-project.org/data-access/user-registration.html

"SHARE-ERIC aims to share the SHARE data as soon as possible with the entire scientific community. Access to the SHARE data is provided free of charge on the basis of a release policy that gives quick and convenient access to all scientific users world-wide after individual registration. All details about the application and registration process can be found below.

SHARE data may be used for scientific research, subject to European Union and national data protection laws and the SHARE Conditions of Use. Furthermore, SHARE-ERIC promises to the participants in the study that the data will not be used for other purposes.

Therefore, any other uses, such as a commercial use of the data, are excluded as matter of principle.

In accordance with the legal requirements and ethical commitments related to the SHARE data collection, applicants have to acknowledge and agree that they will use the SHARE data only for scientific purposes. In this connection, they also have to provide relevant information with regard to their scientific alignment during the registration process."

Acknowledgements:

This paper uses data from SHARE Waves 2 and 3 (SHARELIFE) (DOIs: 10.6103/SHARE.w2.610, 10.6103/SHARE.w3.610), see Börsch-Supan et al. (2013) for methodological details. The SHARE data collection has been primarily funded by the European Commission through FP5 (QLK6-CT-2001-00360), FP6 (SHARE-I3: RII-CT-2006-062193, COMPARE: CIT5-CT-2005-028857, SHARELIFE: CIT4-CT-2006-028812) and FP7

For peer review only - http://bmjopen.bmj.com/site/about/guidelines.xhtml

(SHARE-PREP: N°211909, SHARE-LEAP: N°227822, SHARE M4: N°261982). Additional funding from the German Ministry of Education and Research, the Max Planck Society for the Advancement of Science, the U.S. National Institute on Aging (U01_AG09740-13S2, P01_AG005842, P01_AG08291, P30_AG12815, R21_AG025169, Y1-AG-4553-01, IAG_BSR06-11, OGHA_04-064, HHSN271201300071C) and from various national funding sources is gratefully acknowledged (see www.share-project.org).

We gratefully acknowledge the insightful comments of referees.

Ethics:

An ethics statement related to SHARE/SHARELIFE, the only data on which we rely for this paper, is available at http://www.share-project.org/fileadmin/pdf_documentation/SHARE_ethics_approvals.pdf.

Competing interests:

None to declare

Funding: This project was supported by the Investeringsagenda of the University of Groningen.

Patient and Public Involvement: no patient involvement.

For peer review only

Abstract

Objectives: We aim to study the association of childhood urbanicity with depressive symptoms in late adulthood.

Design, setting & participants: We use linear and logistic regression to analyse data drawn from 20,400 respondents from the Survey on Health, Ageing and Retirement in Europe, a panel dataset incorporating a representative sample of the 50+ population in 13 European countries.

Outcomes & analysis: Childhood urbanicity is determined using self-reports of the respondents' circumstances at age 10, and late-adulthood depression using the EURO-D scale. We condition on circumstances early in life as well as later in life, most importantly late-adulthood urbanicity. We estimate the associations using linear regression models and limited dependent variable models.

Results: A pooled regression of both men and women suggests childhood urbanicity is associated non-monotonically with depression in late adulthood, and particularly apparent for those spending their childhoods in suburban settings. We find that individuals that spend the longest time in their childhood in a suburban home exhibit an average increase in probability of 3.4 (*CI* 1.1—5.7) percentage points in reporting four or more depressive symptoms. The association is robust to the inclusion of a host of household characteristics associated with childhood urbanicity, and is independent of current urbanicity and current income. When broken down by gender, we find some evidence of associations between depressive outcomes and urban living for men, and stronger evidence of such associations with urban and suburban living for women, who exhibit an increase of 5.6 (*CI* 2.2—9.0) percentage points in reporting four or more depressive symptoms.

Conclusions: Our analysis reveals a relationship between childhood urbanicity and depression in late-adulthood. The evidence presented on the nature of this relationship is not straightforward but broadly suggestive of a link, differing by gender, between greater urbanicity and higher levels of depressive symptoms. The life-long nature of this association may potentially inform policy agendas aimed at improving urban & suburban living conditions.

Strengths & limitations of this study

- This is the first paper, to the best of our knowledge, to focus on long-term relationships between childhood urbanicity of residence and late-adulthood depressive outcomes specifically.
- We find evidence pointing towards a link between childhood urbanicity and late adulthood depressive outcomes, and highlight potential pathways for this.
- Due to the administrative structure of our data and the absence of any plausible identifying restrictions for causal effects, we are unable to make strong claims regarding causality.

For peer review only - http://bmjopen.bmj.com/site/about/guidelines.xhtml

Keywords: urbanisation; depression; mental health; lifecourse / childhood circumstances; ageing.

For peer review only

For peer review only - http://bmjopen.bmj.com/site/about/guidelines.xhtml

INTRODUCTION

The increase in prevalence of mental health disorders in the late 20th and early 21st century, along with increased urban dwelling, raises the question of interplay between these two phenomena. While a substantial body of literature, outlined below, examines links between the lived environment and mental health outcomes at various points over the life course, a potential long-term relationship between childhood urbanicity and late-life depression remains less substantially researched.

A World Health Organisation review[1] reports that an estimated 300 million people suffer from depression globally. Associations with further health conditions and wider adverse socioeconomic outcomes are well-explored, with one meta-analysis[2] evidencing the multiplicity of social costs of major depression, such as reduced educational outcomes, adverse labour market and health outcomes, and increases in other health-damaging behaviours.

As of 2014, 54% of the world's population reside in urban areas, with this figure having risen from 30% since 1950 and being set to rise to 66% by 2050[3]. Various pathways have been suggested for links between levels of urbanicity and health outcomes. Vlahov & Galea [4] report that the urban social environment can affect individuals' general health via certain features such as socioeconomic status, crime and violence, presence of marginalised populations and the prevalence of psychological stressors. Diez Roux & Mair[5] further detail potential pathways for links between physical and social aspects of the lived environment and current health outcomes. Compared to living in a rural area, urban dwellers face an elevated amount of environmental stressors on a daily basis which can lead to long-term degradation of their mental well-being. Further detail of links between city dwelling, quality of life and happiness is provided in [6], with aspects of city dwelling such as pollution, monotony of life, and the artificiality of the city suggested as being potentially detrimental to quality of life. A

For peer review only - http://bmjopen.bmj.com/site/about/guidelines.xhtml

Finnish study[7] suggests that this may be due in part to variation in socioeconomic status between rural and urban areas.

Existing literature has generally focused on the relationships between urbanicity and contemporaneous health outcomes at various stages of the life course, with a number of these covering common mental disorders such as depression[8–13]. While the authors caution against drawing causal conclusions, in a broad meta-analysis of the link between urbanicity and mental health disorders, Penkalla & Kohler[14] find links between the degree of urbanicity and mood and anxiety disorders, psychosis, and substance use disorders. The largest single study[15], using administrative data collected on the entire Swedish population of 4.4 million people aged 25-64, finds that those in the top quintile by urbanicity have a 68-77% higher risk of developing psychosis and 12-20% higher risk of developing depression than those in the lowest, with these relationships being stronger for women than men. Oliver[16] discusses potential links between current suburban and urban living and adverse mental health outcomes.

The relationship between childhood urbanicity and depression later in life remains a topic substantially less explored in existing research. While some evidence [17,18] points to relationships between early life urban dwelling and impacts on brain areas associated with negative affect, we believe there to exist only one study[19] to attempt to carry out preliminary investigations into longer-term and later-life relationships that directly use measures of depression. Literature on the life course perspective[20–23] suggests, however, theoretical roles for the impact of conditions throughout an individual's life (in particular of early life conditions) on subsequent health, with this impact likely to vary in particular by gender (as evidenced by theoretical and empirical reviews[24,25]). Various pathways have been proposed to explain the incidence of depression specifically over the lifecycle, with Rudenstine[26] emphasising a role for the impact of early life circumstances.

For peer review only - http://bmjopen.bmj.com/site/about/guidelines.xhtml

Previous studies examining longer-arm relationships between urbanicity and mental ill-health suggest empirical support for these theoretical pathways. One study employing the records of over 2.3 million people born in Sweden between 1967 and 1989[27], finds that the population density of area of residence at age 15 is predictive of subsequent schizophrenia. Kim, Stewart, Shin, Yoon, & Lee[19] use the principal areas that participants lived in during early-life (up to age 20) and mid-life (21-60) to investigate the relationship between the urbanicity of residence at such ages and depression later in life. The study finds links between current urbanicity and current depressive outcomes, and a greater prevalence of depression among women. Individuals who lived their entire life in rural areas are found to have the lowest rates of depression, with similar rates of depression observed among individuals who grew up in rural areas and subsequently lived in urban areas, and those who spent their entire life in urban areas. Lederbogen et al[17], using magnetic resonance imaging to examine brain activity, present evidence of relationships between urban dwelling in childhood and social stress processing. A further study[28] uses German and Chinese data to investigate links between childhood urbanicity and emotional characteristics later in (mostly young) adulthood. These results, drawn from relatively small samples (324 individuals in Germany, 713 in China) provide some evidence suggestive of a relationship with negative emotional traits for Chinese women alone, among whom *lower* symptoms of "fear" are exhibited for those who lived in urban settings in childhood.

In this paper, we seek to investigate specifically the relationship between early-life urban living and the presence of depression in late adulthood. We employ a cross-country European dataset of individuals aged 50 and above in order to investigate such a relationship, using individuals' self-reports of childhood circumstances, and depressive symptoms as measured by the EURO-D questionnaire. Section 2 introduces our data source and variables used in our study. Section 3 discusses the results of our study and its implications. Section 4 concludes.

For peer review only - http://bmjopen.bmj.com/site/about/guidelines.xhtml

METHODS

Data and sample

For the purpose of our study we employ two waves of the Survey of Health, Ageing and Retirement in Europe (SHARE), a pan-European survey containing information on health, socio-economic and social and family networks of individuals that are aged 50 or older in thirteen European countries plus Israel[1]. SHARE consists of six published waves to date, with individuals tracked across successive surveys. The study design and sampling methods, both in general and for the specific waves here employed, are detailed by the study's authors in several publications[29–33]. A declaration relating to ethical approval is available online [34], and no public or patient involvement beyond this took place. We employ the second contemporaneous wave of the study (collected in 2006-7) and SHARELIFE, the third wave of the survey (collected in 2008-9), which focuses on the self-reported life histories of respondents. In total, 25,341 individuals appear in both datasets. After listwise removal of individuals with missing data for individual variables, our final sample stands at 20,400 (9029 men and 11,371 women) individuals[2], and 13,548 (6,154 men and 7,394 women) when control is made for current urbanicity. A summary of item missingness is shown in Table A1. Table 1 shows the summary statistics for the final sample used in the study, broken down by gender and by childhood urbanicity.

Variables

The main dependent variable used in this study is depression, with symptoms measured by the EURO-D scale included in the SHARE questionnaire. The EURO-D is a symptoms scale for depression and has been validated in a cross-European study[35]. The scale covers 12

[1]Israel is, however, not included in our analysis due to its absence from wave 3.
[2]We also use a larger sample (consisting of 24,854 individuals) for our full information maximum likelihood analysis, containing all individuals in both waves of the data with a recorded EURO-D score.

For peer review only - http://bmjopen.bmj.com/site/about/guidelines.xhtml

symptoms: depressed mood, pessimism, suicidality, guilt, sleep, interest, irritability, appetite, fatigue, concentration, enjoyment and tearfulness. The final EURO-D score (between 0 and 12) is calculated from the total number of these symptoms present, with a score of 4 or more found to be an optimal cut-off point for the prediction of depression[35,36]. In addition to a set of models which treat this EURO-D score as the dependent variable in OLS regressions, in a second set of models, we transform this variable into a dichotomous variable that is equal to 1 for EURO-D scores 4 and higher, and 0 for scores strictly below 4.

The main independent variable of interest in our study is the urbanicity of the respondents' childhood homes. In the retrospective SHARELIFE questionnaire, respondents provided information on each dwelling they had lived in since birth, providing the exact year in which they moved, and the type of area in which each dwelling was located. This area type is coded in SHARELIFE with values of 1 ("A big city"), 2 ("The suburbs or outskirts of a big city"), 3 ("A large town"), 4 ("A small town"), 5 ("A rural area or village"). A previous study investigating the recall of other childhood conditions in SHARE [37] presents evidence that individuals recall these in general accurately.

Durations, measured in years, between moving into and out of each dwelling were computed to determine the length that respondents lived in each of these. We then identified the home that respondents lived in for the longest time between birth and the age of 15, and the corresponding area type of that dwelling. In our base case, we include this variable as is, after the exclusion of cases for non-response.

Statistical analysis

We outline here the various statistical methods employed. We estimate our models primarily as a series of ordinary least squares (OLS) and logistic regressions – the former being used for regressions using the EURO-D score as dependent variable and the latter for those using a

For peer review only - http://bmjopen.bmj.com/site/about/guidelines.xhtml

dichotomised EURO-D variable equal to 1 for scores of 4 or more, and to 0 for scores strictly fewer than 4. We also include a full information maximum likelihood (FIML) specification, which allows us to estimate parameters using all available data in the sample under the assumption that observations are missing at random[38,39], as a final robustness check.

We include basic demographic controls of gender and age-period-cohort in all models, as well as allowing for country fixed effects. In summarising the varying social, marital and economic positions of men and women and the potential tendencies of each to respond to adverse life events in different ways, existing literature[24,40] suggests there may be differences both in overall prevalence of depression by gender, and also in relationships between explanatory factors and such mental health outcomes. Furthermore, while all respondents in the SHARELIFE survey are aged above 50, existing literature points to a role for age as a relevant control variable even among this group[41,42]. Although interpretation of the variation explained by age variables in studies such as this is plagued by problems in separately identifying age-period-cohort effects – that is, due to collinearity, identifying these three effects independently is impossible without resorting to additional, untestable, assumptions[43] – we include these variables purely as controls for the combination of all three potential such effects. While for brevity we subsequently refer to these variables as "age" controls, we remain agnostic as to the actual source of variation by age-period-cohort and merely attempt to control for any such relationship. In an initial parsimonious model, we control only for these basic demographic factors.

In further models, we also include additional control variables related to childhood circumstances and characteristics, and contemporaneous log income[3]. Latham[46] reports that childhood disability is significantly associated with later-life depression, a relationship found

[3]We implement this by dividing gross household income by the square root of household size, as per the current method employed by recent OECD publications[44,45].

For peer review only - http://bmjopen.bmj.com/site/about/guidelines.xhtml

to be of an indirect nature through social and health factors such as midlife physical health. To control for such an effect in our model, we introduce self-reported childhood health status, a score between 1 (poor) to 5 (excellent). We also seek to control for childhood socioeconomic circumstances and, to this end, follow a validated method[37] employed in other papers[47,48] employing SHARE, a principal components score, created from a combination of measures of childhood (age 10) socioeconomic status included in SHARELIFE. Table A2 in the Appendix provides the results of our principal component analysis. We use only component 1 to construct the score used in our index of childhood SES, which we divide into deciles in our model. We also add parental presence (split by step- and biological parent) in the childhood home at the age of ten, self-perceived and self-recalled ability at age ten in mathematics and literacy compared to the average and self-reported parental alcohol abuse.

In a final model, we seek to consider the relationship between depression in late adulthood and childhood urbanicity once control is made for current urbanicity at the time at which the SHARE interview was conducted. To this end, we include a variable for current urbanicity coded in an identical way to that in which we code childhood urbanicity, as earlier described.

We also present alternative specifications, changing the dependent variable to the urbanicity of area of residence when aged between 0-5, 6-10, and 11-15 years.

For peer review only - http://bmjopen.bmj.com/site/about/guidelines.xhtml

RESULTS

[Table 1 here]

Out of the 20,400 individuals included in our main sample, 23% recorded a EURO-D score of 4 or above, with an average score of 2.18. We find that women (29%) were almost twice as likely as men (15%) to exhibit a EURO-D score of 4 or above, and this score for women (2.58) was just under one point higher on average than for men (1.68).

[Tables 2-4 here]

Our results suggest that the urbanicity of childhood residence, rather than of current residence, is of primary importance in the relationship between the lived environment and depression. These results also suggest that such a relationship varies by gender, with an F-test of the interactions between gender and childhood urbanicity categories being rejected at $p < 0.0001$. A matrix of individuals' urbanicity of residence in childhood and currently is presented in Table A3.

Our results suggest a complex relationship between level of childhood urbanicity and later life depression. A parsimonious pooled regression (Column 1, Table 2) including both men and women suggests that, compared to individuals living in rural areas or villages, only individuals spending their childhoods in the suburbs of big cities are observed to have significantly worse outcomes in late adulthood. Our model with childhood controls (Column 2, Table 2) suggests that individuals growing up in big cities are observed to have EURO-D scores of 0.0978 ($p < 0.05$) on average worse than those growing up in rural areas or villages. In the same model, individuals growing up in the suburbs of big cities have worse still depressive outcomes as

For peer review only - http://bmjopen.bmj.com/site/about/guidelines.xhtml

measured by the EURO-D score, with this score being on average 0.153 ($p < 0.01$) higher for such individuals. Individuals growing up in large towns are estimated to have a EURO-D score in adulthood on average 0.0760 ($p < 0.10$) higher than those growing up in rural areas. These results are robust to respecification of the model with our age variable modified to, variously, the type of area lived for longest between the ages of 0-5, 6-10, 11-15 (Table A4)[4].

When broken down by gender, these relationships across our models appear to differ substantially. Much stronger results are observed for women than for men. While only the coefficient for having spent childhood in a big city reaches conventional levels of significance for men (Column 4, Table 2), this relationship is only observed for women for those who grew up in the suburbs of a big city (Column 6, Table 2). Men with childhoods spent in a big city are estimated to have a EURO-D score of on average 0.104 ($p < 0.10$) higher than those who grew up in rural areas or villages, while women with suburban childhoods are observed to have a EURO-D score of on average 0.223 ($p < 0.01$) higher than those who grew up in rural areas or villages.

While these may appear to represent small increases, it is instructive to also consider the results of a logit model with presence of a EURO-D score of 4 or more as dependent variable, with these results presented in Table 3 (odds-ratios) and Table 4 (average marginal effects). Such a model again suggests that suburban childhoods are associated with worse depressive outcomes for women, with an average marginal effect estimated for women of 5.58 percentage points in our preferred model.

While we confirm a role for childhood urbanicity in explaining adult urbanicity (with an F-test of the relevance of such variables in an ordered logit model with adulthood urbanicity as the

[4] We also run our analysis incorporating both marital status and self-reported parental mental health conditions. In each case, there is no change to statistical significance and no substantive change to coefficients associated with area type in childhood. Full results are available on request.

For peer review only - http://bmjopen.bmj.com/site/about/guidelines.xhtml

dependent variable being rejected at $p < 0.0001$), the addition of this as an explanatory variable (Columns 3, 5 & 7, Table 2) does not substantially affect our estimated relationship between urbanicity of childhood residence and the individual's EURO-D score in late adulthood[5].

For peer review only

[5] Although there is a reduction in statistical significance (indeed a loss of significance for our big city point estimate), it is worth noting that the addition of current urbanicity reduces our sample size by approximately one third. None of our point estimates are substantially affected by the addition of current urbanicity.

For peer review only - http://bmjopen.bmj.com/site/about/guidelines.xhtml

DISCUSSION

These results are suggestive of a long–term relationship, persisting beyond the age of 50, between levels of childhood urbanicity and depression. The presence of such a relationship is robust to several model specifications, and is most commonly observed for those who grew up in suburban areas. Contra existing literature, we find that increasing levels of contemporaneous urbanicity generally have a small and non-significant negative relationship with both EURO-D score (in our linear regressions) and presence of a EURO-D score of 4 or more (in our logistic regressions). Furthermore, the inclusion of this additional variable does not substantially impact upon the magnitude of our parameter estimates of the relationship between childhood urbanicity and depressive outcomes. These small and statistically insignificant parameter estimates suggest little evidence of a relationship between current urbanicity and the presence of depression in our sample.

With the continued trend of rapid urbanisation and the rising prominence of mental health conditions[1,3], the investigation of links between childhood urbanicity and mental health outcomes is required to help policy makers better understand and design policies to cater to such growing needs. Our study follows in the footsteps of existing literature on urbanicity and mental health but goes on to specifically look at the relationship of urbanicity and depression from a long-term standpoint, as well as being, to our knowledge, the first of this type to consider separate relationships by gender. In contrast to existing studies in the field, our study employs a cross-national sample with a comprehensive and wide range of retrospective data.

In line with literature in the life course perspective emphasising the importance of early life circumstances, we find that spending one's childhood in an urban or suburban area has a positive and significant relationship with the presence of depression in later-life adulthood when a pooled regression is run for the entire sample. This relationship persists even after

For peer review only - http://bmjopen.bmj.com/site/about/guidelines.xhtml

controlling for other socioeconomic, demographic and environmental factors. This relationship appears to be particularly strong for women. Individuals that spend the longest time in their childhood in a suburban home face an average increase in probability of 3.4 percentage points (5.6 points for women) of reporting four or more depressive symptoms on the EURO-D scale during later-life adulthood, a relationship that persists even when control is made for current urbanicity. It is important to acknowledge that the nature of SHARE is such that going beyond establishing correlations in our data is not possible. While we are unable to establish any identifying restrictions for such causal effects in our dataset, the findings here are striking and establish potential pathways for future research. Although SHARE's rich retrospective information, even if based on recall data, provides a rare opportunity to investigate long-term relationships such as this, the use of linked administrative long panel datasets would be preferable. Such datasets would contain more comprehensive information about specific areas of residence and would help to overcome one limitation of our study: that of treating "big city" as one homogeneous item, borne out of our inability to account for the fact that the experience of city life varies both within and between cities. Future research could seek to overcome some of these inherent limitations of our approach, in seeking to establish causal effects, as well as to establish more clearly theoretical pathways for such effects.

For peer review only - http://bmjopen.bmj.com/site/about/guidelines.xhtml
For peer review only

APPENDIX

[Tables A1-A4 here]

For peer review only - http://bmjopen.bmj.com/site/about/guidelines.xhtml

References

1 World Health Organisation. Mental disorders. WHO. 2017.http://www.who.int/mediacentre/factsheets/fs396/en/ (accessed 7 Aug 2017).

2 Kessler RC. The Costs of Depression. *Psychiatr Clin* 2012;**35**:1–14. doi:10.1016/j.psc.2011.11.005

3 United Nations, Department of Economic and Social Affairs, Population Division. *World Urbanization Prospects, the 2014 Revision: Highlights*. 2014.

4 Vlahov D, Galea S. Urbanization, urbanicity, and health. *J Urban Health Bull N Y Acad Med* 2002;**79**:S1–12. doi:10.1093/jurban/79.suppl_1.S1

5 Diez Roux AV, Mair C. Neighborhoods and health. *Ann N Y Acad Sci* 2010;**1186**:125–45. doi:10.1111/j.1749-6632.2009.05333.x

6 Okulicz-Kozaryn A. *Happiness and Place: Why Life is Better Outside of the City*. 1st ed. 2015 edition. Palgrave Pivot 2015.

7 Airaksinen J, Hakulinen C, Elovainio M, *et al.* Neighborhood effects in depressive symptoms, social support, and mistrust: Longitudinal analysis with repeated measurements. *Soc Sci Med* 2015;**136–137**:10–6. doi:10.1016/j.socscimed.2015.04.034

8 Kim M-H, Jung-Choi K, Jun H-J, *et al.* Socioeconomic inequalities in suicidal ideation, parasuicides, and completed suicides in South Korea. *Soc Sci Med* 2010;**70**:1254–61. doi:10.1016/j.socscimed.2010.01.004

9 Maas J, Verheij RA, Groenewegen PP, *et al.* Green space, urbanity, and health: how strong is the relation? *J Epidemiol Community Health* 2006;**60**:587–92. doi:10.1136/jech.2005.043125

10 Newbury J, Arseneault L, Caspi A, *et al.* Why Are Children in Urban Neighborhoods at Increased Risk for Psychotic Symptoms? Findings From a UK Longitudinal Cohort Study. *Schizophr Bull* 2016;**42**:1372–83. doi:10.1093/schbul/sbw052

11 van de Poel E, O'Donnell O, van Doorslaer E. Urbanization and the spread of diseases of affluence in China. *Econ Hum Biol* 2009;**7**:200–16. doi:10.1016/j.ehb.2009.05.004

12 Weich S, Twigg L, Holt G, *et al.* Contextual risk factors for the common mental disorders in Britain: a multilevel investigation of the effects of place. *J Epidemiol Community Health* 2003;**57**:616–21. doi:10.1136/jech.57.8.616

13 Kovess-Masféty V, Alonso J, de Graaf R, *et al.* A European approach to rural-urban differences in mental health: the ESEMeD 2000 comparative study. *Can J Psychiatry Rev Can Psychiatr* 2005;**50**:926–36. doi:10.1177/070674370505001407

14 Penkalla AM, Kohler S. Urbanicity and Mental Health in Europe: A Systematic Review. *Eur J Ment Health* 2014;**9**:163–77.

For peer review only - http://bmjopen.bmj.com/site/about/guidelines.xhtml

15 Sundquist K, Frank G, Sundquist J. Urbanisation and incidence of psychosis and depression: follow-up study of 4.4 million women and men in Sweden. *Br J Psychiatry J Ment Sci* 2004;**184**:293–8.

16 Oliver JE. Mental Life and the Metropolis in Suburban America: The Psychological Correlates of Metropolitan Place Characteristics. *Urban Aff Rev* 2003;**39**:228–53. doi:10.1177/1078087403254445

17 Lederbogen F, Kirsch P, Haddad L, *et al.* City living and urban upbringing affect neural social stress processing in humans. *Nature* 2011;**474**:498–501. doi:10.1038/nature10190

18 Pezawas L, Meyer-Lindenberg A, Drabant EM, *et al.* 5-HTTLPR polymorphism impacts human cingulate-amygdala interactions: a genetic susceptibility mechanism for depression. *Nat Neurosci* 2005;**8**:828. doi:10.1038/nn1463

19 Kim J-M, Stewart R, Shin I-S, *et al.* Lifetime urban/rural residence, social support and late-life depression in Korea. *Int J Geriatr Psychiatry* 2004;**19**:843–51. doi:10.1002/gps.1175

20 Ben-Shlomo Y, Kuh D. A life course approach to chronic disease epidemiology: conceptual models, empirical challenges and interdisciplinary perspectives. *Int J Epidemiol* 2002;**31**:285–93. doi:10.1093/ije/31.2.285

21 Koenen KC, Rudenstine S, Susser E, *et al.*, editors. *A Life Course Approach to Mental Disorders*. Oxford, New York: : Oxford University Press 2013.

22 Kuh D, Ben Shlomo Y, editors. *A Life Course Approach to Chronic Disease Epidemiology*. Second Edition. Oxford, New York: : Oxford University Press 2004.

23 Pudrovska T, Anikputa B. Early-life socioeconomic status and mortality in later life: an integration of four life-course mechanisms. *J Gerontol B Psychol Sci Soc Sci* 2014;**69**:451–60. doi:10.1093/geronb/gbt122

24 Cleary PD. Gender differences in stress-related disorders. In: Barnett RC, Biener L, Baruch GK, eds. *Gender and stress*. New York, NY, US: : Free Press 1987. 39–72.

25 Van de Velde S, Bracke P, Levecque K. Gender differences in depression in 23 European countries. Cross-national variation in the gender gap in depression. *Soc Sci Med 1982* 2010;**71**:305–13. doi:10.1016/j.socscimed.2010.03.035

26 Rudenstine S. Applying a life course perspective to depression. In: Koenen KC, Rudenstine S, Susser E, *et al.*, eds. *A Life Course Approach to Mental Disorders*. Oxford University Press 2013. 88–96. doi:10.1093/acprof:oso/9780199657018.003.0008

27 Sariaslan A, Larsson H, D'Onofrio B, *et al.* Does Population Density and Neighborhood Deprivation Predict Schizophrenia? A Nationwide Swedish Family-Based Study of 2.4 Million Individuals. *Schizophr Bull* 2015;**41**:494–502. doi:10.1093/schbul/sbu105

28 Sindermann C, Kendrick KM, Becker B, *et al.* Does Growing up in Urban Compared to Rural Areas Shape Primary Emotional Traits? *Behav Sci Basel Switz* 2017;**7**. doi:10.3390/bs7030060

For peer review only - http://bmjopen.bmj.com/site/about/guidelines.xhtml

29 Börsch-Supan A. Survey of Health, Ageing and Retirement in Europe (SHARE) Wave 2. 2017. doi:10.6103/SHARE.w2.600

30 Börsch-Supan A. Survey of Health, Ageing and Retirement in Europe (SHARE) Wave 3 - SHARELIFE. 2017. doi:10.6103/SHARE.w3.600

31 Börsch-Supan A, Brugiavini A, Jürges H, *et al. First Results from the Survey of Health, Ageing, and Retirement in Europe (2004-2007) : Starting the longitudinal dimension*. Mannheim: : Mannheim Research Institute for the Economics of Aging 2008. http://www.share-project.org/fileadmin/pdf_documentation/FRB2/FRB2_all_chapters.pdf (accessed 11 Apr 2017).

32 Börsch-Supan A, Brandt M, Hunkler C, *et al.* Data Resource Profile: the Survey of Health, Ageing and Retirement in Europe (SHARE). *Int J Epidemiol* 2013;**42**:992–1001. doi:10.1093/ije/dyt088

33 Börsch-Supan A, Schröder M. Retrospective data collection in the Survey of Health, Ageing and Retirement in Europe. *SHARELIFE Methodol* 2011;**5**.http://www.share-project.org/uploads/tx_sharepublications/FRB-Methodology_feb2011_color-1.pdf#page=6

34 Wolfrum R. Opinion of the Ethics Council of the Max Planck Society on the 'SHARE' Project. 2016.http://www.share-project.org/fileadmin/pdf_documentation/SHARE_ethics_approvals.pdf

35 Prince MJ, Reischies F, Beekman AT, *et al.* Development of the EURO-D scale--a European, Union initiative to compare symptoms of depression in 14 European centres. *Br J Psychiatry* 1999;**174**:330–8. doi:10.1192/bjp.174.4.330

36 Prince MJ, Beekman AT, Deeg DJ, *et al.* Depression symptoms in late life assessed using the EURO-D scale. Effect of age, gender and marital status in 14 European centres. *Br J Psychiatry J Ment Sci* 1999;**174**:339–45.

37 Havari E, Mazzonna F. Can We Trust Older People's Statements on Their Childhood Circumstances? Evidence from SHARELIFE. *Eur J Popul* 2015;**31**:233–57. doi:10.1007/s10680-014-9332-y

38 Schafer JL, Graham JW. Missing data: Our view of the state of the art. *Psychol Methods* 2002;**7**:147–77. doi:10.1037/1082-989X.7.2.147

39 Enders CK. The Performance of the Full Information Maximum Likelihood Estimator in Multiple Regression Models with Missing Data. *Educ Psychol Meas* 2001;**61**:713–40. doi:10.1177/0013164401615001

40 Simon RW. Revisiting the relationships among gender, marital status, and mental health. *AJS* 2002;**107**:1065–96.

41 Keyes M, Legrand LN, Iacono WG, *et al.* Parental Smoking and Adolescent Problem Behavior: An Adoption Study of General and Specific Effects. *Am J Psychiatry* 2008;**165**:1338–44. doi:10.1176/appi.ajp.2008.08010125

For peer review only - http://bmjopen.bmj.com/site/about/guidelines.xhtml

42 Mirowsky J, Ross CE. Age and Depression. *J Health Soc Behav* 1992;**33**:187–205. doi:10.2307/2137349

43 Bell A, Jones K. The impossibility of separating age, period and cohort effects. *Soc Sci Med 1982* 2013;**93**:163–5. doi:10.1016/j.socscimed.2013.04.029

44 Organisation for Economic Co-operation and Development. Divided We Stand: Why Inequality Keeps Rising: An overview of growing income inequalities in OECD countries: main findings. *OECD Publl* Published Online First: 2011.http://www.ingentaconnect.com/content/oecd/16815378/2011/00002011/00000014/8111111ec003

45 Organisation for Economic Co-operation and Development. What are equivalence scales? *OECD Proj Income Distrib Poverty* 2013.

46 Latham K. The 'long arm' of childhood health: linking childhood disability to late midlife mental health. *Res Aging* 2015;**37**:82–102. doi:10.1177/0164027514522276

47 Angelini V, Mierau J. Late-life health effects of teenage motherhood. *Demogr Res* 2018;**39**:1081–104. doi:10.4054/DemRes.2018.39.41

48 Angelini V, Howdon DDD, Mierau JO. Childhood Socioeconomic Status and Late-Adulthood Mental Health: Results from the Survey on Health, Ageing and Retirement in Europe. *J Gerontol B Psychol Sci Soc Sci* Published Online First: 16 March 2018. doi:10.1093/geronb/gby028

For peer review only - http://bmjopen.bmj.com/site/about/guidelines.xhtml

Tables

TABLE 1
Descriptive Statistics (N = 20,400)

Name	All	All	Mean / % Men	Mean / % Women	
Individual Characteristics			(N=9,029)	(N=11,371)	
Male (N, %)	9,029	*44.26*	N/A	N/A	
Age (mean, *sd*)	64.23	*9.43*	64.34	64.15	
Urbanicity of current residence (mean, *sd*)	3.33	*1.41*	3.35	3.31	
Urbanicity of childhood residence (mean, *sd*)	3.70	1.46	3.67	3.71	
Equivalised HH income (000s) (mean, *sd*)	21.57	*30.69*	23.36	20.16	***
Health Status					
Depression (EURO-D)	2.18	*2.15*	1.68	2.58	***
EURO-D score 4+ (N, %)	4,666	*22.87*	*14.67*	*29.38*	***
Childhood SES					
Number of Rooms (mean, *sd*)	3.72	*1.79*	3.73	3.71	
Number of Facilities (mean, *sd*)	2.28	*1.48*	2.28	2.28	
Number of Books (1 = few to 5 = >200 books) (mean, *sd*)	2.13	*1.21*	2.11	2.14	
Occupation of Main Breadwinner (ranked) (mean, *sd*)	2.90	*0.80*	2.90	2.91	
Childhood Characteristics					
Math Performance (1 = low to 5 = high) (mean, *sd*)	3.30	*0.89*	3.39	3.23	***
Language Performance (1 = low to 5 = high) (mean, *sd*)	3.33	*0.87*	3.24	3.41	***
Self-Reported Health (1 = poor to 5 = excellent) (mean, *sd*)	3.97	*1.00*	4.04	3.91	***
Parent heavy drinker (N, %)	1,658	*8.13*	*8.09*	*8.16*	
Biological Father Present (N, %)	18,582	*91.09*	*91.15*	*91.04*	
Biological Mother Present (N, %)	19,639	*96.27*	*3.41*	*3.98*	*
Stepfather Present (N, %)	441	*2.16*	*2.10*	*2.21*	
Stepmother Present (N, %)	322	*1.58*	*1.46*	*1.67*	

Note: */**/*** indicate significant differences at the 10%/5%/1% level based on Student's t-tests of group mean comparisons.

For peer review only - http://bmjopen.bmj.com/site/about/guidelines.xhtml

TABLE 2
Association Between Childhood Urbanicity and Late-Adulthood EURO-D score (OLS coefficients)

		Full Sample		Men	Men	Women	Women
Dependent variable	EURO-D	EURO-D	EURO-D	EURO-D	EURO-D	EURO-D	EURO-D
	(1)	(2)	(3)	(4)	(5)	(6)	(7)
Age 0-15 living environment (omitted: rural area or village)							
Big city	0.0474	0.0978**	0.117**	0.104*	0.0755	0.0937	0.151*
	(0.0432)	(0.0440)	(0.0566)	(0.0582)	(0.0740)	(0.0641)	(0.0836)
Suburbs or outskirts of a big city	0.117**	0.153***	0.156**	0.0563	0.0355	0.223***	0.253**
	(0.0560)	(0.0562)	(0.0704)	(0.0751)	(0.0913)	(0.0812)	(0.105)
Large town	0.0208	0.0760*	0.0977*	0.0653	0.0812	0.0834	0.124
	(0.0433)	(0.0436)	(0.0558)	(0.0583)	(0.0726)	(0.0631)	(0.0826)
Small town	-0.0275	0.0136	0.0153	0.0818	0.114*	-0.0471	-0.0791
	(0.0412)	(0.0410)	(0.0518)	(0.0552)	(0.0683)	(0.0590)	(0.0759)
Male	- 0.887***	- 0.846***	- 0.864***				
	(0.0286)	(0.0287)	(0.0355)				
Current living environment (omitted: rural area or village)							
Big city			-0.0850		-0.0755		-0.0634
			(0.0603)		(0.0800)		(0.0883)
Suburbs or outskirts of a big city			-0.0528		0.0267		-0.115
			(0.0588)		(0.0759)		(0.0879)
Large town			0.0299		-0.0670		0.136*
			(0.0541)		(0.0715)		(0.0792)
Small town			-0.0840		-0.0868		-0.0669
			(0.0514)		(0.0671)		(0.0760)

For peer review only - http://bmjopen.bmj.com/site/about/guidelines.xhtml

Additional childhood controls & contemporaneous income	NO	YES	YES	YES	YES	YES	YES
Observations	20,400	20,400	13,548	9,029	6,154	11,371	7,394

*Notes: Constant not reported. */**/*** indicate statistical significance at the 10%/5%/1% level, standard errors between brackets.*

All models contain controls for age-period-cohort and country fixed effects. Childhood controls consist of self-reported and self-recalled health status, a principal components score for deprivation, self-perceived and self-recalled numeracy and literacy at age 10, and self-reported parental alcohol abuse

Column 1 displays the estimation results from regressing the EURO-D depression score on a set of living conditions when the respondent was aged 0 – 15. Column 2 adds various childhood & contemporaneous controls and Column 3 controls for current living conditions. Columns 4 & 5 and 6 & 7 repeat the specifications of columns 2 & 3 for men and women separately, respectively.

For peer review only

TABLE 3

Association Between Childhood Urbanicity and Late-Adulthood Depression incidence (Logit, odds ratios)

	Full Sample			Men	Men	Women	Women
	(1)	(2)	(3)	(4)	(5)	(6)	(7)
Age 0-15 living environment (omitted: rural area or village)							
Big city	1.045	1.121**	1.100	1.129	1.021	1.117	1.147
	(0.0566)	(0.0634)	(0.0797)	(0.109)	(0.127)	(0.0783)	(0.103)
Suburbs or outskirts of a big city	1.176**	1.235***	1.217**	1.039	0.990	1.334***	1.350***
	(0.0810)	(0.0876)	(0.108)	(0.132)	(0.152)	(0.115)	(0.148)
Large town	1.036	1.109*	1.087	1.084	1.047	1.120*	1.116
	(0.0556)	(0.0615)	(0.0765)	(0.105)	(0.127)	(0.0761)	(0.0976)
Small town	0.996	1.045	1.057	1.106	1.143	1.014	1.007
	(0.0504)	(0.0540)	(0.0685)	(0.0993)	(0.125)	(0.0643)	(0.0812)
Male	0.399***	0.410***	0.416***				
	(0.0149)	(0.0157)	(0.0193)				
Current living environment (omitted: rural area or village)							
Big city			0.947		0.927		0.980
			(0.0716)		(0.121)		(0.0916)
Suburbs or outskirts of a big city			0.984		1.071		0.945
			(0.0737)		(0.132)		(0.0890)
Large town			1.052		0.915		1.155*
			(0.0705)		(0.106)		(0.0961)
Small town			0.871**		0.825*		0.906
			(0.0565)		(0.0908)		(0.0732)
Additional childhood controls & contemporaneous income	NO	YES	YES	YES	YES	YES	YES
Observations	20,400	20,400	13,548	9,029	6,154	11,371	7,394

For peer review only - http://bmjopen.bmj.com/site/about/guidelines.xhtml

*Notes: Constant not reported. */**/*** indicate statistical significance at the 10%/5%/1% level, standard errors between brackets.*

All models contain controls for age-period-cohort and country fixed effects. Childhood controls consist of self-reported and self-recalled health status, a principal components score for deprivation, self-perceived and self-recalled numeracy and literacy at age 10, and self-reported parental alcohol abuse

Column 1 displays the estimation results from regressing the EURO-D depression score on a set of living conditions when the respondent was aged 0 – 15. Column 2 adds various childhood & contemporaneous controls and Column 3 controls for current living conditions. Columns 4 & 5 and 6 & 7 repeat the specifications of columns 2 & 3 for men and women separately, respectively.

For peer review only

For peer review only - http://bmjopen.bmj.com/site/about/guidelines.xhtml

TABLE 4

Association Between Childhood Urbanicity and Late-Adulthood Depression incidence (Logit, average marginal effects)

	Full Sample			Men	Men	Women	Women
	(1)	(2)	(3)	(4)	(5)	(6)	(7)
Age 0-15 living environment (omitted: rural area or village)							
Big city	0.00717	0.0181**	0.0151	0.0144	0.00240	0.0208	0.0261
	(0.00878)	(0.00903)	(0.0116)	(0.0117)	(0.0147)	(0.0133)	(0.0173)
Suburbs or outskirts of a big city	0.0268**	0.0340***	0.0319**	0.00443	-0.00118	0.0558***	0.0586***
	(0.0117)	(0.0118)	(0.0147)	(0.0148)	(0.0179)	(0.0172)	(0.0220)
Large town	0.00569	0.0162*	0.0132	0.00944	0.00546	0.0212*	0.0209
	(0.00868)	(0.00881)	(0.0112)	(0.0115)	(0.0144)	(0.0129)	(0.0167)
Small town	-0.000625	0.00683	0.00875	0.0118	0.0163	0.00253	0.00129
	(0.00806)	(0.00806)	(0.0102)	(0.0107)	(0.0136)	(0.0117)	(0.0150)
Male	-0.148***	-0.141***	-0.140***				
	(0.00580)	(0.00583)	(0.00714)				
Current living environment (omitted: rural area or village)							
Big city			-0.00870		-0.00923		-0.00388
			(0.0120)		(0.0157)		(0.0178)
Suburbs or outskirts of a big city			-0.00255		0.00866		-0.0108
			(0.0120)		(0.0157)		(0.0178)
Large town			0.00823		-0.0107		0.0282*
			(0.0109)		(0.0139)		(0.0163)
Small town			-0.0216**		-0.0225*		-0.0185
			(0.0101)		(0.0128)		(0.0151)

For peer review only

Additional childhood controls & contemporaneous income	NO	YES	YES	YES	YES	YES	YES
Observations	20,400	20,400	13,548	9,029	6,154	11,371	7,394

*Notes: Constant not reported. */**/*** indicate statistical significance at the 10%/5%/1% level, standard errors between brackets.*

All models contain controls for age-period-cohort and country fixed effects. Childhood controls consist of self-reported and self-recalled health status, a principal components score for deprivation, self-perceived and self-recalled numeracy and literacy at age 10, and self-reported parental alcohol abuse.

Column 1 displays the estimation results from regressing the EURO-D depression score on a set of living conditions when the respondent was aged 0 – 15. Column 2 adds various childhood & contemporaneous controls and Column 3 controls for current living conditions. Columns 4 & 5 and 6 & 7 repeat the specifications of columns 2 & 3 for men and women separately, respectively.

For peer review only

TABLE A1

Item missingness: summary (all individuals in both waves, n=25,341)

Variable	Missing count	Present count
EURO-D	487	24,854
Childhood urban residence	1,715	23,626
Age	716	24,625
Childhood maths performance	859	24,482
Childhood language performance	927	24,414
Childhood self-reported health	189	25,152
Parent heavy drinker	103	25,238
Deprivation score	1,172	24,169
Biological Father Present	506	24,835
Biological Mother Present	506	24,835
Stepfather Present	506	24,835
Stepmother Present	506	24,835
Equivalised HH income	444	24,897
Current urban residence	8,882	16,459

For peer review only

TABLE A2

Principal component analysis for childhood SES

	Component			
	1st	2nd	3rd	4th
Number of Rooms	0.4073	0.8637	-0.2968	-0.0111
Number of Books (grouping)	0.5651	-0.1752	0.2367	0.7707
Occupation of Main Breadwinner (ranked)	-0.4758	0.4695	0.7042	0.2394
Number of Facilities	0.537	-0.0546	0.6	-0.5905

For peer review only

TABLE A3

Urbanicity of childhood residence and of current residence (all values percentages)

		Current residence					
		1	2	3	4	5	Total
Childhood residence	1	37.65	21.28	15.13	11.31	14.63	100
	2	17.19	39.64	14.03	14.84	14.30	100
	3	14.39	16.20	38.87	16.81	13.73	100
	4	11.14	12.62	20.40	38.10	17.75	100
	5	9.31	9.05	15.80	21.98	43.85	100
	Total	15.23	15.04	19.74	21.89	28.11	100

Key

1: A big city

2: The suburbs or outskirts of a big city

3: A large town

4: A small town

5: A rural area or village

For peer review only

TABLE A4

Association Between Stage of Childhood Urbanicity and Late-Adulthood Depression Outcomes

	OLS			Logit, average marginal effects		
	(1)	(2)	(3)	(4)	(5)	(6)
Age	0-5	6-10	11-15	0-5	6-10	11-15
Childhood living environment (omitted: rural area or village)						
Big city	0.0913**	0.104**	0.124***	0.0193**	0.0215**	0.0234***
	(0.0437)	(0.0440)	(0.0439)	(0.00889)	(0.00897)	(0.00893)
Suburbs or outskirts of a big city	0.173***	0.159***	0.194***	0.0234**	0.0209*	0.0152
	(0.0577)	(0.0562)	(0.0554)	(0.0117)	(0.0114)	(0.0114)
Large town	0.0706	0.0781*	0.0977**	0.00527	0.00831	0.00729
	(0.0439)	(0.0437)	(0.0434)	(0.00915)	(0.00912)	(0.00905)
Small town	-0.0119	0.0165	0.0187	-0.00171	0.00242	-0.00300
	(0.0410)	(0.0410)	(0.0409)	(0.00874)	(0.00874)	(0.00875)
Male	-0.845***	-0.845***	-0.845***	-0.121***	-0.121***	-0.121***
	(0.0287)	(0.0287)	(0.0287)	(0.00583)	(0.00583)	(0.00583)
Observations	20,400	20,400	20,400	20,400	20,400	20,400

*Notes: Constant not reported. */**/*** indicate statistical significance at the 10%/5%/1% level, standard errors between brackets.*

All models contain controls for age-period-cohort, country fixed effects and additional childhood controls & contemporaneous income.

For peer review only

STROBE 2007 (v4) checklist of items to be included in reports of observational studies in epidemiology*
Checklist for cohort, case-control, and cross-sectional studies (combined)

Section/Topic	Item #	Recommendation	Reported on page #
**Title and abstract**	1	(*a*) Indicate the study's design with a commonly used term in the title or the abstract	3
		(*b*) Provide in the abstract an informative and balanced summary of what was done and what was found	3
**Introduction**			
Background/rationale	2	Explain the scientific background and rationale for the investigation being reported	4-6
Objectives	3	State specific objectives, including any pre-specified hypotheses	6
**Methods**			
Study design	4	Present key elements of study design early in the paper	7-10
Setting	5	Describe the setting, locations, and relevant dates, including periods of recruitment, exposure, follow-up, and data collection	7
Participants	6	(*a*) *Cohort study*—Give the eligibility criteria, and the sources and methods of selection of participants. Describe methods of follow-up<br>*Case-control study*—Give the eligibility criteria, and the sources and methods of case ascertainment and control selection. Give the rationale for the choice of cases and controls<br>*Cross-sectional study*—Give the eligibility criteria, and the sources and methods of selection of participants	7
		(*b*) *Cohort study*—For matched studies, give matching criteria and number of exposed and unexposed<br>*Case-control study*—For matched studies, give matching criteria and the number of controls per case	n/a
Variables	7	Clearly define all outcomes, exposures, predictors, potential confounders, and effect modifiers. Give diagnostic criteria, if applicable	7-10
Data sources/ measurement	8*	For each variable of interest, give sources of data and details of methods of assessment (measurement). Describe comparability of assessment methods if there is more than one group	*7-10*
Bias	9	Describe any efforts to address potential sources of bias	8-9
Study size	10	Explain how the study size was arrived at	7 (also Table A3)
Quantitative variables	11	Explain how quantitative variables were handled in the analyses. If applicable, describe which groupings were chosen and why	7-10
Statistical methods	12	(*a*) Describe all statistical methods, including those used to control for confounding	7-10
		(*b*) Describe any methods used to examine subgroups and interactions	11
		(*c*) Explain how missing data were addressed	7, 9
		(*d*) *Cohort study*—If applicable, explain how loss to follow-up was addressed<br>*Case-control study*—If applicable, explain how matching of cases and controls was addressed	7, 9

For peer review only

		Cross-sectional study—If applicable, describe analytical methods taking account of sampling strategy	
		(*e*) Describe any sensitivity analyses	9-10
**Results**			
Participants	13*	(a) Report numbers of individuals at each stage of study—eg numbers potentially eligible, examined for eligibility, confirmed eligible, included in the study, completing follow-up, and analysed	7
		(b) Give reasons for non-participation at each stage	Table A3
		(c) Consider use of a flow diagram	n/a
Descriptive data	14*	(a) Give characteristics of study participants (eg demographic, clinical, social) and information on exposures and potential confounders	7
		(b) Indicate number of participants with missing data for each variable of interest	Table A3
		(c) *Cohort study*—Summarise follow-up time (eg, average and total amount)	
Outcome data	15*	*Cohort study*—Report numbers of outcome events or summary measures over time	
		Case-control study—Report numbers in each exposure category, or summary measures of exposure	
		Cross-sectional study—Report numbers of outcome events or summary measures	7, 21
Main results	16	(*a*) Give unadjusted estimates and, if applicable, confounder-adjusted estimates and their precision (eg, 95% confidence interval). Make clear which confounders were adjusted for and why they were included	22-27
		(*b*) Report category boundaries when continuous variables were categorized	7-8
		(*c*) If relevant, consider translating estimates of relative risk into absolute risk for a meaningful time period	n/a
Other analyses	17	Report other analyses done—eg analyses of subgroups and interactions, and sensitivity analyses	24-27
**Discussion**			
Key results	18	Summarise key results with reference to study objectives	14
Limitations	19	Discuss limitations of the study, taking into account sources of potential bias or imprecision. Discuss both direction and magnitude of any potential bias	15
Interpretation	20	Give a cautious overall interpretation of results considering objectives, limitations, multiplicity of analyses, results from similar studies, and other relevant evidence	15
Generalisability	21	Discuss the generalisability (external validity) of the study results	15
**Other information**			
Funding	22	Give the source of funding and the role of the funders for the present study and, if applicable, for the original study on which the present article is based	n/a

*Give information separately for cases and controls in case-control studies and, if applicable, for exposed and unexposed groups in cohort and cross-sectional studies.

Note: An Explanation and Elaboration article discusses each checklist item and gives methodological background and published examples of transparent reporting. The STROBE checklist is best used in conjunction with this article (freely available on the Web sites of PLoS Medicine at http://www.plosmedicine.org/, Annals of Internal Medicine at http://www.annals.org/, and Epidemiology at http://www.epidem.com/). Information on the STROBE Initiative is available at www.strobe-statement.org.

For peer review only
